# Impedance Spectroscopy Study of Solid Co(II/III) Redox Mediators Prepared with Poly(Ethylene Oxide), Succinonitrile, Cobalt Salts, and Lithium Perchlorate for Dye-Sensitized Solar Cells

**DOI:** 10.3390/polym18010142

**Published:** 2026-01-04

**Authors:** Ravindra Kumar Gupta, Ahamad Imran, Aslam Khan, Muhammad Ali Shar, Khalid M. Alotaibi, Idriss Bedja, Abdullah Saleh Aldwayyan

**Affiliations:** 1 King Abdullah Institute for Nanotechnology, King Saud University, Riyadh 11451, Saudi Arabia; aimran@ksu.edu.sa (A.I.); aslamkhan@ksu.edu.sa (A.K.); mashar@ksu.edu.sa (M.A.S.); khalid.m@ksu.edu.sa (K.M.A.); 2 Department of Chemistry, College of Science, King Saud University, Riyadh 11451, Saudi Arabia; 3 Department of Optometry, College of Applied Medical Sciences, King Saud University, Riyadh 11433, Saudi Arabia; bedja@ksu.edu.sa; 4 Department of Physics and Astronomy, College of Science, King Saud University, Riyadh 11451, Saudi Arabia; dwayyan@ksu.edu.sa

**Keywords:** DSSC, solid polymer electrolytes, plasticizers, electrical conductivity, FT-IR spectroscopy, XPS

## Abstract

Countries like Saudi Arabia receive abundant sunshine with exceptionally high solar irradiance. High temperatures in desert regions and the sunray angle dependence of solar modules are some of the key challenges of conventional solar cells. Dye-sensitized solar cells present a compelling alternative with the simple cell design and use of non-toxic materials without angle dependence, but their performance hinges on the solid redox mediators used for dye regeneration. These mediators must have an electrical conductivity (σ_25°C_) of more than 10^−4^ S cm^−1^ with an activation energy of less than 0.3 eV for device application. Our work focused on novel solid Co(II/III) redox mediators using cobalt complexes and LiClO_4_ in different matrices: pure PEO (an abbreviation for poly(ethylene oxide) with its redox mediator as M1), a [PEO–SN] blend (M2A and M2B with ethylene oxide to lithium ions molar ratio of 112.9 and 225.8, respectively), and pure SN (an abbreviation for succinonitrile with its redox mediator as M3). Impedance spectroscopy was the key technique, showing M1 and M2 behave like a mediator explainable with an (R_1_–C)-type circuit, while M3 is explainable with an (R_1_ − [R_2_‖C])-type circuit. M3 achieved the highest value of σ_25°C_ with 2 × 10^−3^ S cm^−1^, while M1 had the lowest σ_25°C_, 3 × 10^−5^ S cm^−1^. M2 achieved an optimal balance with σ_25°C_ of 4 × 10^−4^ S cm^−1^ (M2A) and 1.5 × 10^−4^ S cm^−1^ (M2B). M2 exhibited a remarkably low pseudo-activation energy of 0.042 eV and a Vogel–Tammann–Fulcher behavior ideal for consistent performance across temperatures. In contrast, M1 and M3 showed higher Arrhenius-type activation energies (>0.74 eV) in their solid states. These results correlated with those of the XRD, FT-IR spectroscopy, XPS, SEM, DSC, and TGA analyses. Ultimately, the [PEO–SN] blend emerges as a robust matrix, enabling the combination of high conductivity and low activation energy needed for a durable device in harsh environments.

## 1. Introduction

Polymers are macromolecules formed by the chemical bonding of repeating structural units known as monomers. The characteristics of polymers are influenced by the specific monomers utilized, their configuration, and the extent of polymerization. They may exhibit flexibility, rigidity, elasticity, heat resistance, or biodegradability, which contribute to their versatility. Polymers play a crucial role in numerous applications, including energy technologies. Poly(ethylene oxide), or PEO, is a polymer widely utilized in dye-sensitized solar cells (DSSCs). For a review on polymer-based DSSCs, see references [1,2,3,4,5,6,7,8,9,10]. PEO has [CH_2_–CH_2_–O] as a monomer that has a size similar to the cationic size. High-molecular-weight PEO generates a self-standing film exhibiting thermal stability up to 200 °C, thereby ensuring the safety of the DSSC in the elevated temperature conditions prevalent in Gulf countries. PEO is an environmentally friendly and biodegradable polymer with a low material cost. The advantageous properties of PEO include (i) a dielectric constant ranging from 5 to 8 and a Gutmann donor number of 22, which facilitate the dissociation and complexation of ionic salts; (ii) the segmental motion of linear chains and the presence of ethereal oxygens, which enhance ion transport; and (iii) optimal solvation of cations via its monomer size [11,12,13]. PEO is combined with a mixture of ionic salts to create a redox couple that is mobile within the solid PEO matrix. This substance is identified as a solid polymer electrolyte. In a dye-sensitized solar cell, this compound is referred to as a solid redox mediator (SRM) due to its role in mediating ions for the regeneration of photosensitive dye molecules [1]. An SRM must exhibit an electrical conductivity ($\sigma_{25\;{}^{\circ}C}$) exceeding 10^−4^ S cm^−1^ and an activation energy ($E_{a}$) below 0.3 eV to ensure optimal performance in a DSSC [14]. However, PEO–ionic salts redox mediators exhibit electrical conductivity of less than 10^−4^ S cm^−1^ at 25 °C. This results from high PEO crystallinity. The crystallinity of PEO is diminished, and its recrystallization is suppressed through the addition of a plasticizer, which can be a low molecular weight polymer, an ionic liquid, a biopolymer, nanoparticles, or a plastic crystal [15,16,17,18,19,20,21,22,23,24,25,26,27,28,29].

Gupta et al. [30] blended a plastic crystal, succinonitrile (or SN), with PEO in an equal weight fraction and used it as a solid matrix to synthesize a solid redox mediator. The SRMs had MI (M = Li or K) and I_2_ ionic salts to produce a $I^{-}/I_{3}^{-}$ redox-couple [25,26,27,28,29]. The solid redox mediators with a ${Co}^{2+}/{Co}^{3+}$ redox-couple had Co[bpy]_3_(TFSI)_2_ and Co[bpy]_3_(TFSI)_3_ ionic salts [31,32]. The bpy and TFSI are short forms of tris-(2,2′-bipyridine) and bis(trifluoromethyl) sulfonylimide, respectively. Both bpy and TFSI are beneficial for improving the electrical transport properties of redox mediators. The cobalt-based SRMs included a lithium salt, either LiTFSI [31] or LiCF_3_SO_3_ [32], where LiTFSI produced a better SRM [33]. The lithium salt provides Li^+^ ions to intercalate into the TiO_2_ nanoparticles for increasing the rate of electron’s injection from excited dye molecules to the working electrode (mesoporous TiO_2_ layer) [28,34]. The composition of cobalt-based SRMs was based on the optimized composition reported by Mathew et al. [35], which is 0.25M Co[bpy]_3_(TFSI)_2_, 0.06M Co[bpy]_3_(TFSI)_3_, and 0.1M LiTFSI in acetonitrile. We replaced the acetonitrile with the succinonitrile. Succinonitrile is known to exhibit the plastic crystal phase with increased concentration of trans isomers between −38 °C (phase transition temperature) and 58 °C (melting temperature), which allows high molecular diffusivity [36,37]. A dielectric constant of 55 at 25 °C and 62.6 at 58 °C, a molar enthalpy of 139.7 kJ mol^−1^, and a donor number of 14 kcal mol^−1^ with a low melting temperature made succinonitrile a beneficial solid solvent [38]. The cyano radical also provides cation transport. The waxy nature of succinonitrile is an added advantage in preparing a homogeneous self-standing film with improved electrical transport parameters. The blend-based ${Co}^{2+}/{Co}^{3+}$ redox mediators are over 90% transparent after 350 nm, while the $I^{-}/I_{3}^{-}$ redox mediators are over 90% transparent after 500 nm [27,31,32]. In addition, the use of iodine makes the $I^{-}/I_{3}^{-}$ redox mediators highly corrosive and reactive with the adjacent metallic components as well as the sealants, which reduces the shelf-life of the DSSC [1,39,40,41,42]. The relatively higher open-circuit voltage of the DSSCs makes the ${Co}^{2+}/{Co}^{3+}$ redox mediators better than the $I^{-}/I_{3}^{-}$ redox mediators [42]. Table S1 (Supplementary Information) presents the [poly(ethylene oxide)–succinonitrile] blend-based $I^{-}/I_{3}^{-}$ and ${Co}^{2+}/{Co}^{3+}$ redox mediators with electrical transport parameters for comparison [25,26,27,28,29,31,32,33]. This table demonstrated that blend-based SRMs exhibit the Vogel−Tamman−Fulcher-type behavior with σ_25°C_ > 10^−4^ S cm^−1^ and pseudo-activation energy < 0.1 eV. These SRMs are thermally stable up to 125 °C.

Electrical conductivity and activation energy are essential parameters of the ion transport phenomenon of ionic conductors for device applications [11,12]. Impedance spectroscopy (IS) illustrates the ion transport phenomenon through Bode and Nyquist plots, which help determine the ion transport parameters [43]. The present paper reports an impedance spectroscopy study of a new solid Co(II/III) redox mediator (M2) prepared with PEO, SN, Co[bpy]_3_(TFSI)_2_, Co[bpy]_3_(TFSI)_3_, and lithium perchlorate (LiClO_4_) for dye-sensitized solar cell application. The M2A and M2B had a molar ratio of ethylene oxide to lithium ions of 112.9 and 225.8, respectively. Figure S1 (Supplementary Information) illustrates the chemical structure of the ingredients. We have also reported an impedance spectroscopy study of M1 and M3 with PEO and SN, respectively, as a solid matrix for comparison. Lithium perchlorate is the third in the series after the LiTFSI [31] and LiCF_3_SO_3_ [32]. This ionic salt is selected because it is a well-known ionic solid for batteries [44]. A liquid electrolyte with LiClO_4_ in an equimolar solvent mixture, ethylene carbonate, and dimethyl carbonate, exhibited ionic conductivity and ionic mobility higher than those of liquid electrolytes with LiTFSI or LiCF_3_SO_3_. LiClO_4_ has high thermal/electrochemical stability. Some other characteristics are: LiTFSI > LiCF_3_SO_3_ > LiClO_4_ for the molecular weight; LiTFSI ≥ LiClO_4_ > LiCF_3_SO_3_ for the dissociation constant; LiTFSI ≥ LiClO_4_ > LiCF_3_SO_3_ for the ion solvation number; LiTFSI < LiClO_4_ < LiCF_3_SO_3_ for the donor number, and TFSI^−^ > ${ClO}_{4}^{-}$ > ${CF}_{3}{SO}_{3}^{-}$ for the anionic size. The impedance spectroscopy results were elucidated through XRD, FT-IR spectroscopy, XPS, SEM, DSC, and TGA studies. This paper has established that the blend of poly(ethylene oxide) and succinonitrile is an excellent choice as a solid matrix to achieve improved electrical conductivity and activation energy while retaining the thermal stability.

## 2. Materials and Methods

Table S2 illustrates the chemicals used for the synthesis of M1, M2, and M3. The chemicals were used without further purification. Table S3 portrays the chemical composition of SRMs, which are based on those reported earlier by Mathew et al. [35] and Gupta et al. [31,32]. M1 and M2 were synthesized following the solution-cast approach. The homogeneous solutions of M1 and M2 were prepared in acetonitrile (20 mL) under stirring at 65 °C for 48 h. The solutions were then cast on Teflon Petri dishes and left for drying slowly in a nitrogen gas-filled desiccator for more than two weeks. The desiccator was evacuated and left for two days. This approach led to the production of self-standing films for M1 and M2. As mentioned earlier, succinonitrile is melted nearly at 58 °C. Therefore, ionic salts were added into melted succinonitrile and stirred for 24 h to prepare M3.

Table S4 describes the methodology used for characterizing the SRMs in detail. It includes the conventional techniques such as impedance spectroscopy (IS, Palmsens, model PalmSens4, Houten, The Netherlands), Fourier-transform infrared spectroscopy (FT-IR, Perkin Elmer, Waltham, MA, USA), X-ray photoelectron spectroscopy (XPS, Photoelectron, Tokyo, Japan), X-ray diffractometry (XRD, Bruker, model D2 Phaser, Karlsruhe, Germany), scanning electron microscopy (SEM, JEOL, Tokyo, Japan), differential scanning calorimetry (DSC, Mettler-Toledo, Schwerzenbach, Switzerland), and thermogravimetric analysis (TGA, Mettler-Toledo, Schwerzenbach, Switzerland).

## 3. Results and Discussion

**Figure 1:**
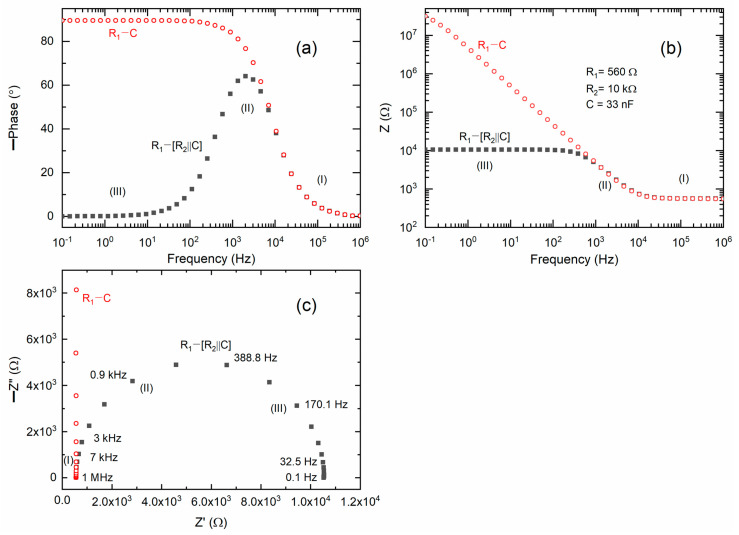
(**a**,**b**) Bode plots of phase angle and impedance, respectively, for ideal circuits (1) R_1_ − (R_2_‖C) and (2) R_1_ − C. (**c**) Nyquist plots for ideal circuits (1) and (2).

Figure 1a presents Bode plots illustrating the phase angle for the ideal circuits (1) R_1_ − (R_2_‖C) and (2) R_1_ − C, and are similar to those reported earlier [43]. Circuit (1) is indicative of an electrolyte situated between non-blocking electrodes, whereas circuit (2) is representative of a blocking electrode configuration. The Bode plot for circuit (1) exhibited a Gaussian-type peak in the mid-frequency region (II) and displayed nearly flat curves in the high (I) and low (III) frequency regions. Circuit (2) generates a Bode plot resembling a step ladder, characterized by a flat curve in region I, a ramp with a negative slope in region II, and a flat curve in region III. Figure 1b presents Bode plots of impedance for the ideal circuits designated as (1) and (2). Circuit (1) exhibited a step ladder-type pattern, characterized by a flat curve in region I, a ramp with a slope of −1 in region II, and a flat curve in region III. Circuit (2) exhibited a flat curve in region I and a ramp with a slope of −1 in regions II and III. Figure 1c presents Nyquist plots for circuits (1) and (2). Circuit (1) produced a semicircle that intersects the real axis (Z′) in regions I and III. In contrast, circuit (2) generates a vertical line with an intercept solely in region I, attributable to the capacitive effect.

**Figure 2:**
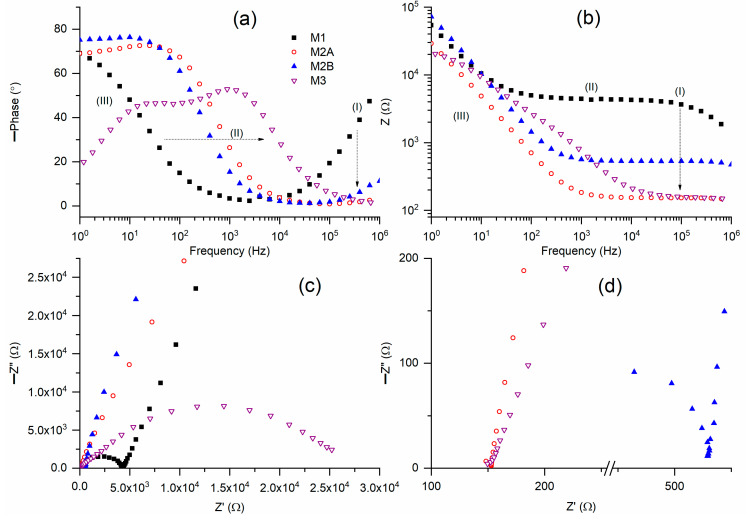
(**a**,**b**) Bode plots of phase angle and impedance, respectively, for solid redox mediators M1, M2A, M2B, and M3 at a temperature of 25 °C. (**c**) Nyquist plots for solid redox mediators M1, M2A, M2B, and M3. (**d**) High-frequency region of Nyquist plots for M2 and M3.

Figure 2a presents Bode plots of phase angle for solid redox mediators M1, M2A, M2B, and M3 at a temperature of 25 °C. M1 exhibited a broad U-shaped curve, characterized by a circuit (2)-type ramp in region III and a positively sloped curve in region I. M2 displayed characteristics consistent with circuit (2), specifically a flat curve in region I, a ramp in region II, and a flat curve in region III. M3 exhibited a curve analogous to that of circuit (1), albeit with two distinct peaks. This pattern likely arises from the plastic crystal characteristics of succinonitrile, which includes both mobile ions and succinonitrile molecules [37]. The ramp exhibits a frequency hierarchy of M1 < M2B < M2A < M3 at a phase angle of 30°. The ramp of M2 is positioned between those of M1 and M3, likely attributable to the equal weight fraction of PEO and SN. In regions I and III, M2A exhibited a lower phase angle compared to M2B. Figure 2b presents Bode plots of impedance for M1, M2A, M2B, and M3 at approximately 25 °C. M1 and M2 presented the Bode plots for circuit (2). M1 exhibited a flat curve in regions I and II, and a ramp in region III, whereas M2 displayed a flat curve in region I and a ramp extending across regions II and III. M3 exhibited a flat curve in region I, a ramp in regions II and III, and another flat curve in region III, as illustrated in Figure 8b, which will be discussed subsequently. The impedance values in region I exhibited the following order: M1 > M2B > M2A ≈ M3. Figure 2c presents Nyquist plots for M1, M2A, M2B, and M3 at approximately 25 °C. Figure 2d presents the Nyquist curves for M2 and M3 in region I. M1 contained a semicircle in region I and a slanted line in regions II and III. The former results from ionic diffusion, whereas the latter arises from the blocking electrode effect. M2A featured a slanted line, whereas M2B included a partial semicircle in addition to a slanted line. M3, as a pure succinonitrile-based redox mediator, exhibited a complete semicircle in regions II and III, along with a partial semicircle in region I. The Nyquist plots exhibit similarities to the previously reported solid Co(II/III) redox mediators [31,32,33]. The intercept in region I represents the bulk resistance of the redox mediator. The bulk resistance, along with the thickness and area of the SRM film (cf. Table S4), determined the electrical conductivity at 25 °C.

Table 1 presents the σ_25°C_ values of M1, M2A, M2B, and M3 for comparative analysis. M1 exhibited a conductivity of 3 × 10^−5^ S cm^−1^, significantly higher than that of pure PEO (σ_25°C_ ≈ 10^−10^ S cm^−1^). This demonstrated that PEO effectively dissociates the cobalt and lithium salts. This is further facilitated by the elevated dissociation constant and ion solvation number of LiClO_4_. The segmental mobility of PEO chains, ethereal oxygens, plasticizers (large anions and cobalt ions), and optimum cation solvation all contributed to a reduction in PEO crystallinity, facilitating rapid ion transport. The electrical conductivity of M1 is significantly greater than the σ_25°C_ values of pure PEO-based solid Co(II/III) redox mediators previously reported [31,32,33]. The DSC analysis, elaborated later, demonstrated the creation of a solid solution-type scenario due to ${ClO}_{4}^{-}$ ions, which contributed to higher conductivity. M2A had a conductivity of nearly 4 × 10^−4^ S cm^−1^, while M2B demonstrated a little lower conductivity of around 1.5 × 10^−4^ S cm^−1^. The observations indicated that M2 possesses σ_25°C_-values an order of magnitude greater than M1 and four orders of magnitude higher than the PEO−SN blend (σ_25°C_ ≈ 10^−8^ S cm^−1^). The σ_25°C_ value of M2 is equivalent to those made with the PEO-SN blend (cf. Table S1). The improvement of conductivity is attributable to the advantageous properties of succinonitrile for ion transport, including its plasticizing characteristics and the presence of cyano radicals. These advantageous conditions enable M3 to attain a high electrical conductivity of 2 × 10^−3^ S cm^−1^ at 25 °C, which is an order of magnitude more than that of M2. One can observe that σ_25°C_ values of the blend-based redox mediators (M2A and M2B) fell between those of the redox mediators made using the blend’s constituents (M1 and M3).

**Figure 3:**
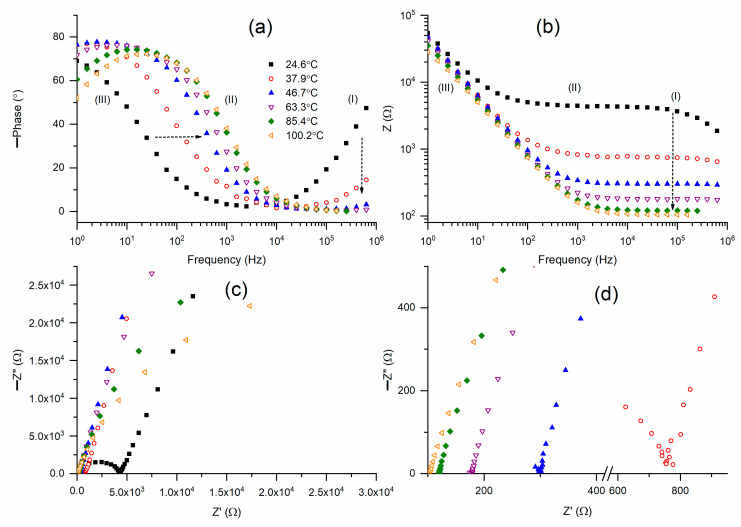
(**a**,**b**) Bode plots of phase angle and impedance, respectively, for solid redox mediator M1 at different temperatures. (**c**) Nyquist plots for M1 at different temperatures. (**d**) High-frequency region (I) of Nyquist plots.

The nature of the redox mediator and its activation energy must be ascertained by measuring σ with temperature [12]. Bode plots of the phase angle for M1 at temperatures between 25 °C and 100 °C are displayed in Figure 3a. As previously stated, the mediator at 25 °C showed a wide U-shaped curve that encompassed regions I–III. At 25 °C, the tilted curve in region I became almost flat at 47 °C, and subsequently it became totally flat. Likewise, at 25 °C, the circuit (2)-type ramp in region III underwent a major shift to region II until it reached 47 °C, after which it underwent a slow shift. In region III, M1 at 25 °C lacked a circuit (2)-type flat curve. This flat was created by raising the temperature to 47 °C. A slanted curve with an increasing positive slope developed as the temperature rose further. According to this figure, M1 at 47 °C exhibited a Bode curve that resembled the Bode pattern of an ideal circuit (2), and 47 °C is a phase transition temperature. Figure 3b revealed comparable findings. Bode graphs of impedance for M1 at temperatures ranging from 25 °C to 100 °C are shown in Figure 3b. M1 (25 °C) partially displayed the Bode pattern of circuit (2), with a ramp in region III and a broad flat curve in regions I and II. At 47 °C, the flat curve’s width drastically shrank along with the impedance value. Similar, albeit negligible, decreases in width and impedance values were seen with further temperature increases. The Nyquist plots of M1 at various temperatures are shown in Figure 3c. For comparison, the Nyquist curves in region I are shown in Figure 3d. Region I of M1 at 25 °C showed a semicircle, while regions II and III showed a slanted line. As previously stated, the blocking electrode action causes the slanted line to form, but the ionic diffusion phenomenon causes the semicircle. The semicircle formation linked to the slanted line decreased as the temperature rose to 47 °C. The semicircle was eliminated by further temperature increases, leaving only a slanted line. The bulk resistance of the M1 is shown by the intercept of the slanted line in the Z’-axis. Figure 3c,d demonstrated that bulk resistance decreased as temperature increased, and this decline was rapid up to 47 °C, resulting in a fast increase in conductivity (σ) up to 47 °C.

**Figure 4:**
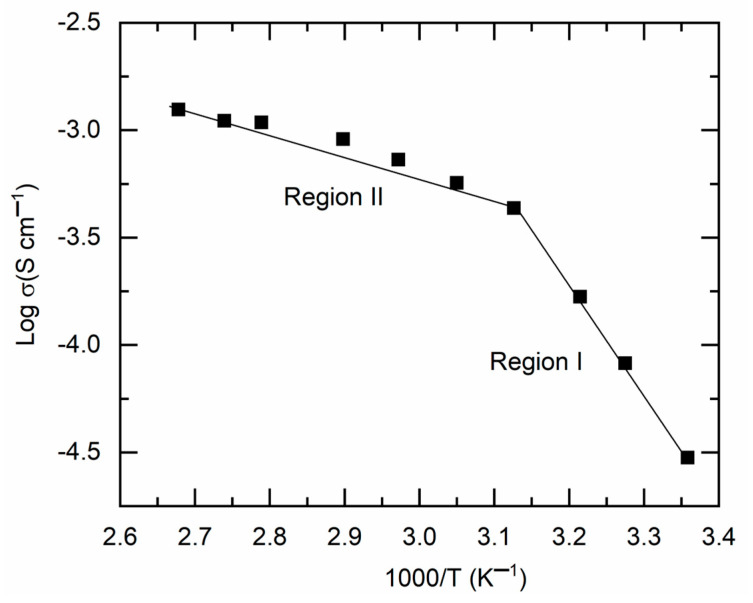
$Log\;\sigma -T^{-1}$ plot for M1 with semi-crystalline phase (region I) and amorphous phase (region II).

Figure 4 presents a $log\;\sigma -T^{-1}$ plot for M1. This image illustrated a linear increase in log σ-value with rising temperature (or a decline in $T^{-1}$), characterized by two distinct slopes, divided at ~47 °C. A similar pattern has been previously noted for a solid redox mediator, synthesized in the same manner using PEO, Co salts, and LiX (where X = TFSI or CF_3_SO_3_) (cf. Table S1). The DSC curve, to be detailed further, indicated that M1 begins to transition from a semi-crystalline phase to an amorphous phase at ~47 °C. The temperature intervals preceding and succeeding 47 °C are designated as region I (semi-crystalline phase) and region II (amorphous phase), respectively. The linearity of the $log\;\sigma -T^{-1}$ curve indicates Arrhenius-type behavior of M1, suggesting that ions are thermally activated for transport. This pattern is expressed as $\sigma =\sigma_{o}{exp[-E}_{a}/k_{B}T]$, where $k_{B}$ is the Boltzmann constant and $\sigma_{o}$ represents the pre-exponential factor [11,12,13]. The linear curve fitting yielded the slope and intercept, which correspond to the activation energy and pre-exponential factor, respectively, as shown in Table 1 for comparison. M1, characterized by a semi-crystalline phase in region I, exhibited elevated values of $E_{a}$ (0.99 eV) and $\sigma_{o}$ (2.1 × 10^12^ S cm^−1^), rendering it unsuitable for DSSCs. Conversely, M1 exhibited low levels of $E_{a}$ (0.2 eV) and $\sigma_{o}$ (1.62 S cm^−1^) in region II.

**Figure 5:**
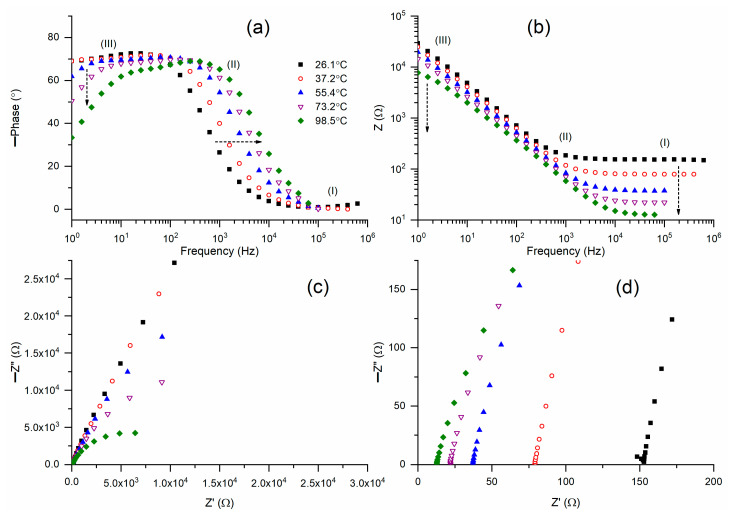
(**a**,**b**) Bode plots of phase angle and impedance, respectively, for solid redox mediator M2A at different temperatures. (**c**) Nyquist plots for M2A at different temperatures. (**d**) High-frequency region (I) of Nyquist plots.

Figure 5a displays Bode plots of phase angle for M2A across a temperature range of 26 °C to 99 °C. M2A (26.1 °C) displayed a step ladder-type pattern of circuit (2), except for a slightly slanted flat curve in region I. An increase in temperature resulted in the flattening of the curves in region I, while region II’s ramps were progressively shifted to higher frequencies, and the inclination of the flat curves in region III was enhanced. Figure 5b presents Bode plots of impedance for M2A over a temperature range of 26 °C to 99 °C. M2A (26.1 °C) exhibited a Bode pattern for circuit (2), characterized by a flat curve in region I and partially in region II, followed by a ramp in region III. An increase in temperature progressively diminished the width of the flat curve and the impedance. Figure 5c presents Nyquist plots of M2A across various temperatures. Figure 5d illustrates the high-frequency region of the Nyquist curves. M2A (26.1 °C) exhibited a partial semicircle followed by an inclined line. The temperature rise eliminated the semicircle, leaving only the slanted lines. The intercept of the slanted line on the real axis indicates the bulk resistance, which in turn determines the electrical conductivity. Figure 5d demonstrates a reduction in bulk resistance, indicating an enhancement in electrical conductivity as temperature rises.

**Figure 6:**
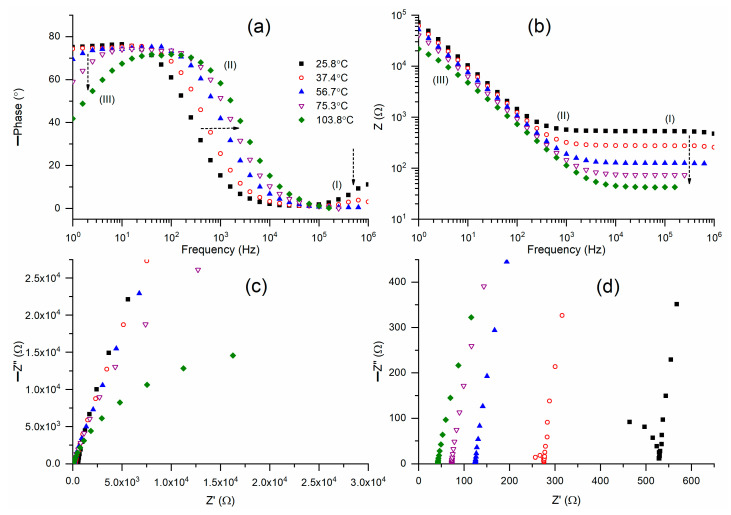
(**a**,**b**) Bode plots of phase angle and impedance, respectively, for solid redox mediator M2B at different temperatures. (**c**) Nyquist plots for M2B at different temperatures. (**d**) High-frequency region (I) of Nyquist plots.

Figure 6a depicts Bode plots for the phase angle of M2B across a temperature range from 26 °C to 104 °C. M2B exhibited Bode curves analogous to those of M2A. M2B (25.8 °C) displayed a step ladder-type pattern of circuit (2), except for a slanted flat curve observed in region I. An increase in temperature progressively diminished the inclination to zero in region I, while simultaneously elevating the inclination in region III. The ramp of region II gradually shifted to higher frequencies. Figure 6b presents Bode plots of impedance for M2B at various temperatures. The Bode curves exhibited a trend consistent with that observed for M2A. M2A (25.8 °C) exhibited a Bode plot for circuit (2) characterized by a flat curve in regions I and II, alongside a ramp in region III. An increase in temperature resulted in a gradual decrease in both the width of the flat curve and the impedance value. Consequently, the Nyquist curve at 25.8 °C, as illustrated in Figure 6c,d, exhibits a slanted line in regions II and III, accompanied by a partial semicircle in region I. M2B at 37.4 °C exhibited a similar trend with a decreased bulk resistance value. An additional temperature rise resulted in the disappearance of the semicircle and the emergence of lines, accompanied by a reduction in the bulk resistance value.

**Figure 7:**
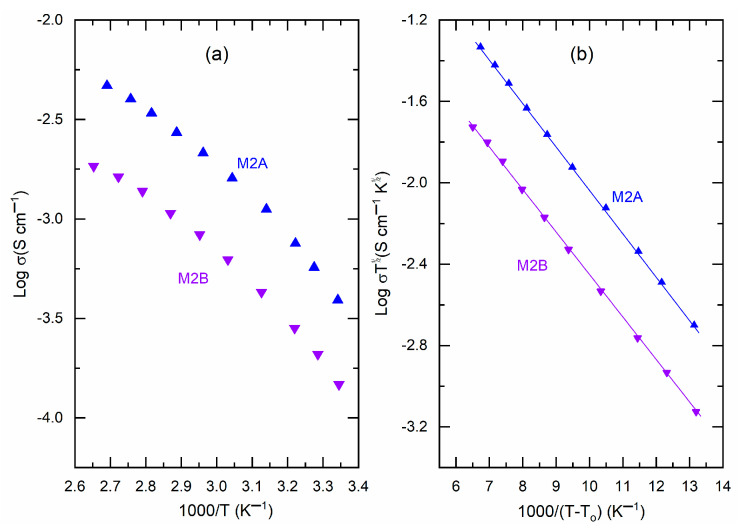
(**a**) $Log\;\sigma -T^{-1}$ plots and (**b**) Vogel−Tamman−Fulcher plots for M2A and M2B.

The temperature-dependent values of bulk resistance and, consequently, the electrical conductivity for M2A and M2B produced $log\;\sigma -T^{-1}$ plots, as shown in Figure 7a. This figure illustrated a non-linear reduction in log σ-value as $T^{-1}$ increases. The curves exhibited low curvature, indicating the presence of amorphous domains that facilitate the random motion of PEO chains and segmental motion. Vogel, Tamman, and Fulcher articulated this phenomenon as $\sigma =\sigma_{o}T^{-1/2}exp[-E_{a}/k_{B}(T-T_{o})$ [11,12,13]. A linear plot of $log\;\sigma T^{1/2}$ against $({T-T_{o})}^{-1}$, as shown in Figure 7b, provides the slope and intercept, which correspond to the pseudo-activation energy (*E_a_*) and the pre-exponential factor ($\sigma_{o}$), respectively. Table 1 presents the values of *E_a_* and $\sigma_{o}$ for M2A and M2B for comparative analysis. M2A and M2B exhibited comparable pseudo-activation energy values of 0.042 eV. Nonetheless, M2A exhibited $\sigma_{o}$ ≈ 1.26 S cm^−1^, which was marginally lower than the 2.23 S cm^−1^ observed for M2B, suggesting a somewhat superior ion transport environment in M2A. Table 1 and Table S1 indicate that σ_25°C_ exceeds 10^−4^ S cm^−1^ and *E_a_* is less than 0.1 eV for various PEO–SN blend-based solid redox mediators. This establishes the PEO–SN blend as a superior option for synthesizing fast ion-conducting redox mediators for DSSC applications. This results from various advantageous characteristics of poly(ethylene oxide) and succinonitrile, as previously discussed.

**Figure 8:**
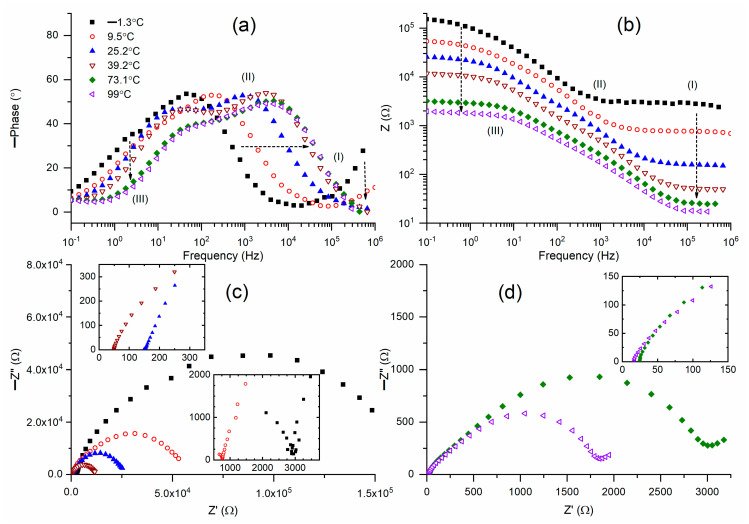
(**a**,**b**) Bode plots of phase angle and impedance, respectively, for solid redox mediator M3 at different temperatures. (**c**) Nyquist plots for M3 at −1.3, 9.5, 25.2, and 39.2 °C. (**d**) Nyquist plots for M3 at 73.1 and 99 °C. Insets, high-frequency region (I) of Nyquist plots.

Figure 8a presents Bode plots illustrating the phase angle of M3 across a temperature spectrum from −1 °C to 99 °C. M3 (−1.3 °C) had a broad Gaussian peak pattern of circuit (1) at 45.2 Hz (the interface of regions II and III), accompanied by a weak shoulder peak at 1.7 Hz (region III). This also exhibited a sloped curve in region I. An elevation in temperature to 39.2 °C diminished the slope of region I’s curve to zero. This was associated with a shift in the peak position from 45 Hz to 3070 Hz (region II) and a displacement of the shoulder peak from 1.7 Hz to 32.5 Hz (region III). This also positively shifted the ramp (slope = −1). An additional temperature rise did not significantly affect the peak position of region II or the shoulder peak position of region III, along with the corresponding ramps. Figure 8b presents Bode graphs of the impedance of M3 across a temperature range of −1 °C to 99 °C. M3 (−1.3 °C) exhibited a Bode curve resembling the step ladder configuration of circuit (1). The flat curve entirely encompassed region I and partially included region II. The ramp ascended to area III, succeeded by a brief horizontal curve in region III. An elevation in temperature to 39.2 °C reduced the width of the flat curve confined solely to region I. In contrast, the width of the flat curve expanded to encompass region III. These alterations were also coupled with a large reduction in impedance in regions I and III, and a moderate reduction in region II. An additional rise in temperature from 39.2 °C to 99 °C resulted in a negligible reduction in impedance. Flat curves also encompassed regions I and III, entirely without variation in width. Figure 8c,d illustrates the Nyquist plots of M3 across temperatures ranging from −1 °C to 99 °C. Insets show the high-frequency region (I) of M3. M3(−1.3 °C) illustrated a semicircle in areas II and III, together with a partial semicircle in region I, which is presented as an inset in Figure 8c. An elevation in temperature progressively diminished the diameter of the semicircle in regions II and III and eliminated the partial semicircle in region I. The insets of Figure 8c,d depict the intersection of the semicircle with the real axis at various temperatures. The intercept, indicative of bulk resistance, diminished as the temperature rose.

**Figure 9:**
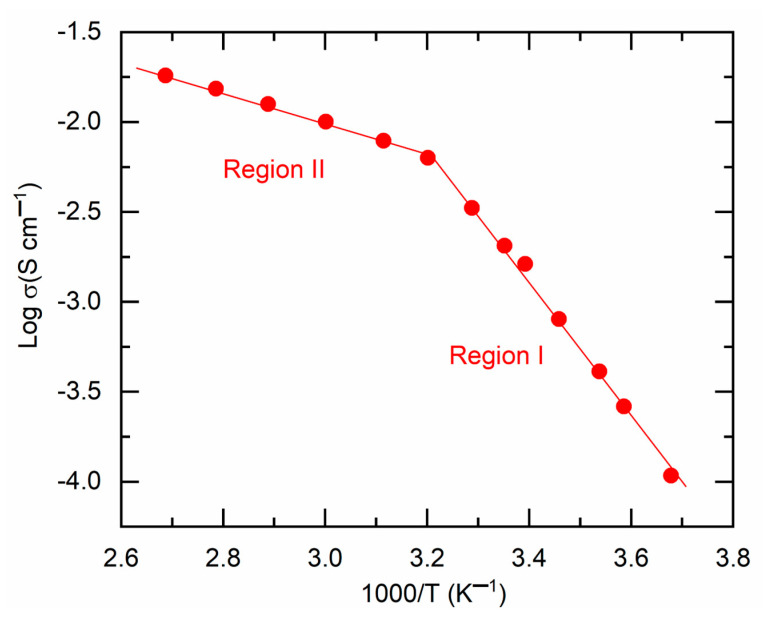
$Log\;\sigma -T^{-1}$ plot for M3 with solid-state phase (region I) and liquid phase (region II).

Figure 9 illustrates the $log\;\sigma -T^{-1}$ plot for M3, delineating the solid-state phase (region I) and the liquid phase (region II). Both regions had a linear decline in log σ-value as $T^{-1}$ increased, indicating the Arrhenius-type behavior of M3. This process is characterized by several succinonitrile-based solid $I^{-}/I_{3}^{-}$ and ${Co}^{2+}/{Co}^{3+}$ redox mediators [31,32,45]. The linear curve fitting yields the slope and intercept, which correspond to the activation energy and pre-exponential factor, respectively, as shown in Table 1 for comparison. M3 exhibited elevated values of $E_{a}$ (0.74 eV) and $\sigma_{o}$ (6 × 10^9^ S cm^−1^) in region I due to the solid state nature of the mediator. Due to its liquid state, M3 exhibited low values of $E_{a}$ (0.17 eV) and $\sigma_{o}$ (4.5 S cm^−1^). M3 demonstrated a conductivity of 2 × 10^−3^ S cm^−1^; nevertheless, it displayed elevated levels of activation energy and pre-exponential factor in region I, rendering it inappropriate for DSSC applications.

**Figure 10:**
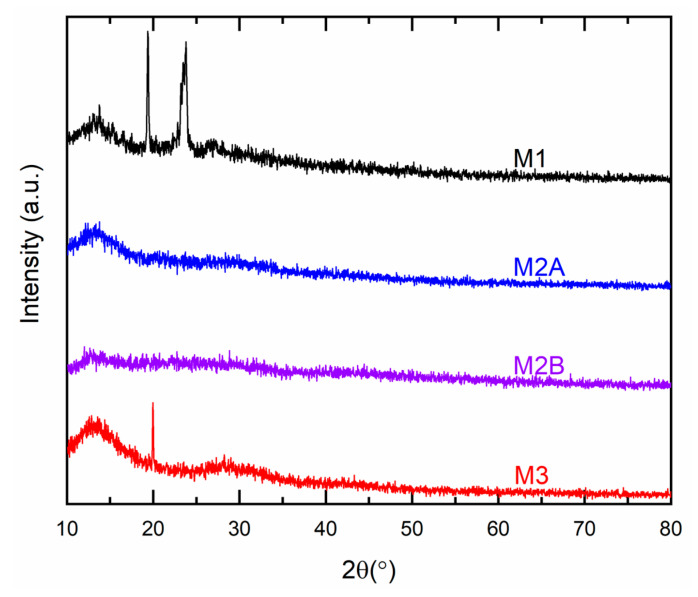
XRD curves for solid redox mediators M1, M2A, M2B, and M3 at 25 °C.

Figure 10 illustrates the XRD patterns for M1, M2A, M2B, and M3. M1 exhibited two minor peaks at 19.4° and 23.8°, aligning with the PEO matrix peaks at 19.2° and 23.3° [30]. An increase in 2θ-values of M1 indicated a reduction in the spacing between polymeric chains to accommodate larger ions of cobalt and lithium salts [46]. A reduction in peak intensity indicated a decline in PEO crystallinity [30]. M2A and M2B did not display any peak corresponding to PEO or SN. Previous reports indicate that in the presence of ions, polymeric chains of PEO function as an impurity, disrupting the crystalline structure of succinonitrile [47]. M3 exhibited a minor peak at 2θ ≈ 20°, surpassing the characteristic peak of succinonitrile at 19.7°. This alteration signified a disruption associated with the presence of large ions [37]. The redox mediators exhibited no reflection peaks of ionic salts, indicating complete dissolution-complexation of ionic salts by the matrix [31,32,33].

**Figure 11:**
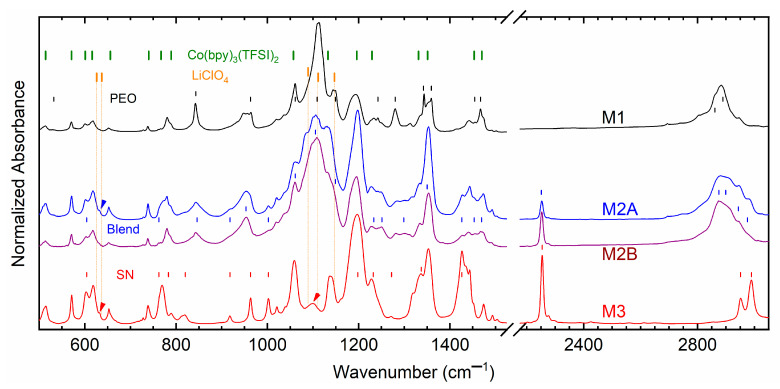
FT-IR spectra for solid redox mediators M1, M2A, M2B, and M3 at 25 °C. Thick vertical lines for vibrational modes of Co(bpy)_3_(TFSI)_2_ and LiClO_4_. Thin vertical lines for vibrational modes of a solid matrix, PEO, (PEO–SN) blend, or SN.

Figure 11 shows FT-IR spectra of M1, M2A, M2B, and M3. The vertical lines indicate vibrational modes of the matrix and ionic salts for comparison. Figure S2 depicts FT-IR spectra of ionic salts. As mentioned in our previous reports [31,32,33], Co(bpy)_3_(TFSI)_2_ or Co(bpy)_3_(TFSI)_3_ comprises vibrational modes of bpy and TFSI^−^. Therefore, the observed vibrational frequencies of the cobalt salt are assigned accordingly. LiClO_4_ displayed a doublet at 626 cm^−1^ due to the stretching mode of free ${ClO}_{4}^{-}$ with a shoulder at 637 cm^−1^ due to stretching mode of coordinated ${ClO}_{4}^{-}$ [48]. A triplet was observed at 1089(s), 1111, and 1147 cm^−1^ due to ν_ClO4_. For M1 and M2, the triplet landed on the PEO’s territory. Assignments for the solid matrices, PEO, SN, and PEO–SN blend have been reproduced from our earlier report [26,27]. Table S5 summarizes the observed vibrational frequencies of solid redox mediators, matrices, and ionic salts for comparative analysis.

M1 exhibited a strong PEO–${ClO}_{4}^{-}$ interaction through a shift in several modes, ν_s,COC_, ν_CC_, δ_a,CH2_, and ν_s,CH2_, which are highlighted in Table S5 with red color. The ν_s,CH2_ mode at 2889 cm^−1^ of PEO shifted to 2884 cm^−1^ for M1. The PEO–bpy interaction was observed at 780 and 1442 cm^−1^. M2A and M2B had the blend–${ClO}_{4}^{-}$ inteaction with a shift in δ_CH2_, ν_s,COC_, δ_a,CH2_, and ν_s,CH2_, which are marked with blue color. The ν_s,CH2_ mode shifted from 2875 cm^−1^ of PEO to 2881 cm^−1^ for M2A and 2876 cm^−1^ for M2B. The ν_s,CH2_ shift was higher for M2A, which resulted in a better environment for ion transport. M2A also exhibited the ‘free’ ν_ClO4_ peak at 632 cm^−1^, which is marked by an arrow. The bpy interacted with the blend as evinced at 780 and 1441 cm^−1^ for M2A and 780 and 1463 cm^−1^ for M2B. M3 displayed the least interaction through SN–bpy, only with a shift in δ_CH2_, which is marked by green color. As portrayed by an arrow, M3 had slightly displaced ν_ClO4_ peaks at 632 cm^−1^ (free) and 1099 cm^−1^. This study indicated that the interaction followed the order M1 > M2B > M2A > M3 at 25 °C. This order is, therefore, the converse of the order of electrical conductivity. These findings are also corroborative with the results of the XPS study, which has been discussed below.

**Figure 12:**
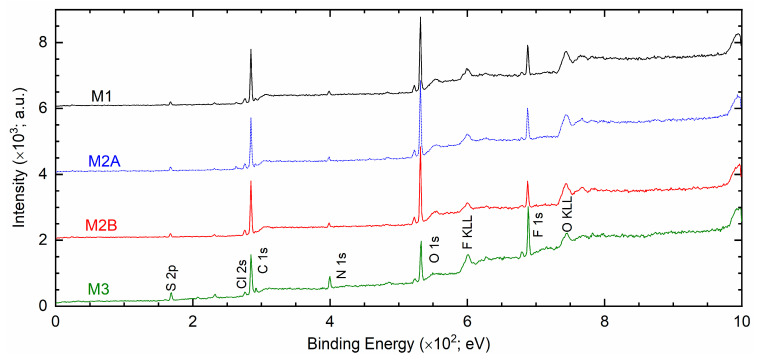
XPS survey spectra of solid redox mediators M1, M2A, M2B, and M3.

The matrix-salt interaction can also be demonstrated by the XPS study because of the involvement of core levels of an element, which depends on the environment [49,50,51]. Figure 12 portrays XPS survey spectra of solid redox mediators M1, M2A, M2B, and M3. The spectra exhibited peaks of S 2p, Cl 2s, C 1s, N 1s, O 1s, and F 1s elements. The peak intensities of PEO-based redox mediators M1, M2A, and M2B were nearly identical. The C 1s, O 1s, and F 1s peaks were strong, and the S 2p, Cl 2s, and N 1s peaks were weak, revealing the dominance of the PEO matrix in an interaction with the ions of the salts. The SN-based mediator M3 exhibited 2.3, 2.8, and 1.6 times higher intensities for S 2p, N 1s, and F 1s peaks, respectively, relative to those of M1. The Cl 2s, C 1s, and O 1s peaks had lower intensities. The survey spectra clearly demonstrated two distinctive groups of solid redox mediators, PEO-like and succinonitrile-like mediators, as observed earlier by Bode plots of phase angle (cf. Figure 2a).

**Figure 13:**
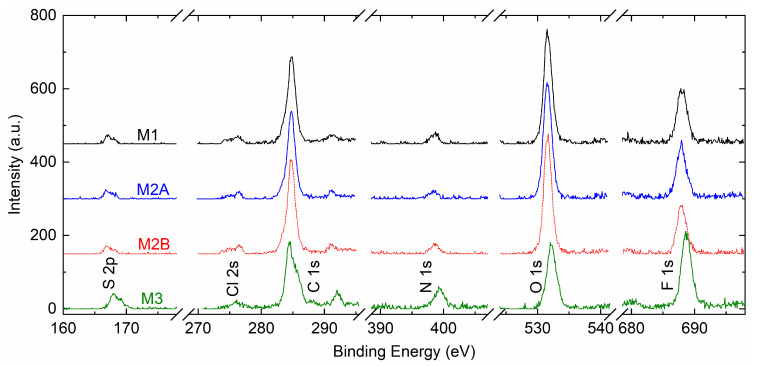
XPS spectra of S 2p, Cl 2s, C 1s, N 1s, O 1s, and F 1s elements of redox mediators M1, M2A, M2B, and M3.

**Figure 14:**
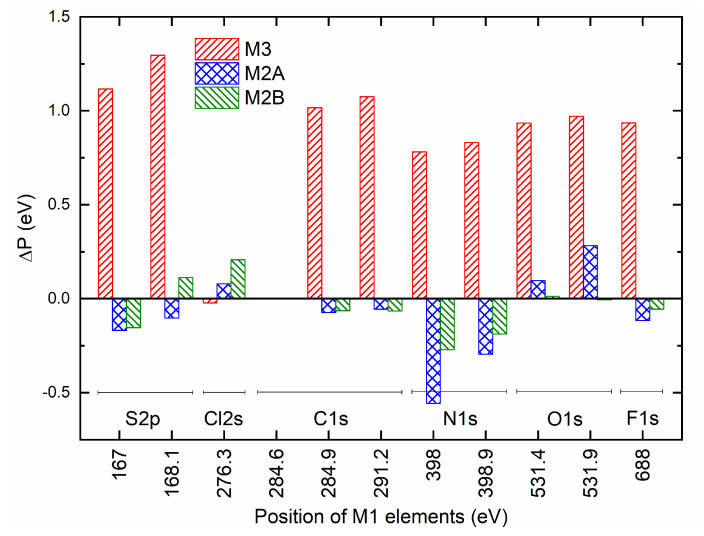
A change in position (ΔP) of elements of a solid redox mediator M2A, M2B, or M3 relative to that of M1.

**Figure 15:**
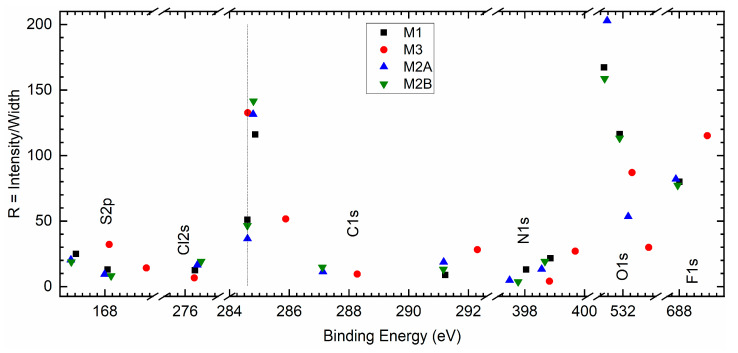
A plot of ratio (R) with peak position for different elements of solid redox mediators M1, M2A, M2B, and M3.

A precise collection of spectra for S 2p, Cl 2s, C 1s, N 1s, O 1s, and F 1s elements of redox mediators M1, M2A, M2B, and M3 is illustrated in Figure 13. As observed earlier in survey spectra, the peak intensities of the S 2p, Cl 2s, C 1s, N 1s, O 1s, and F 1s elements are nearly equal for M1, M2A, and M2B. These PEO-based redox mediators possessed strong peaks for C 1s, O 1s, and F 1s elements with an order of O 1s > C 1s > F 1s. These peaks were also strong for the succinonitrile-based redox mediator M3. However, intensities of C 1s and O 1s were less, and the intensity of F 1s was more than those of M1. To quantify a shift in position (ΔP) of M2A, M2B, and M3 relative to M1 and a dimensionless ratio R (intensity/width), the spectra of elements were smoothened, baselined, and fitted. The fitting was based on our previous work [33]. Figure S3 exhibits the best fit of spectra for the S 2p element of M1, M2A, M2B, and M3. These spectra portrayed a small S 2p peak because of the –SO_2_− group of TFSI^□^ at ≈167 eV for the spin of 3/2, associated with a shoulder peak at ≈168.1 eV for the spin of ½. The best fit of spectra for Cl 2s, C 1s, N 1s, O 1s, and F 1s elements is illustrated and detailed in Figures S4–S8, respectively. Figure 14 portrays a shift in position (ΔP) of elements of a solid redox mediator M2A, M2B, or M3 relative to that of M1. M3 exhibited a large positive shift for all elements, except for the Cl 2s element. This is probably indicative of less interaction between succinonitrile and cations in M3, resulting in the best conducting environment. M2A and M2B depicted a small negative shift for S 2p, C 1s, N 1s, and F 1s elements and a small positive shift for Cl 2s and O 1s elements. However, the magnitude of the shift was higher for M2A. Therefore, M2A probably has less matrix-ion interaction as compared with M2B, resulting in better electrical conductivity. The magnitude of the average of the ΔP can be sequenced as M1 < M2B < M2A < M3. Figure 15 depicts the ratio (R) with binding energy for S 2p, Cl 2s, C 1s, N 1s, O 1s, and F 1s elements of redox mediators M1, M2A, M2B, and M3. In general, the PEO-based redox mediators M1, M2A, and M2B appeared in a bunch, revealing two distinctive groups of solid redox mediators: PEO-like and succinonitrile-like mediators. The M3 ratio was higher for the S 2p, C 1s, N 1s, and F 1s elements, revealing less interaction of succinonitrile with the cations. M2A exhibited a higher ratio for O 1s, providing better ion transport through a less interactive environment. M3 also showed a small peak at ≈288.2 eV for the cyano group. This cyano peak appeared at 287.1 eV for M2A and M2B.

**Figure 16:**
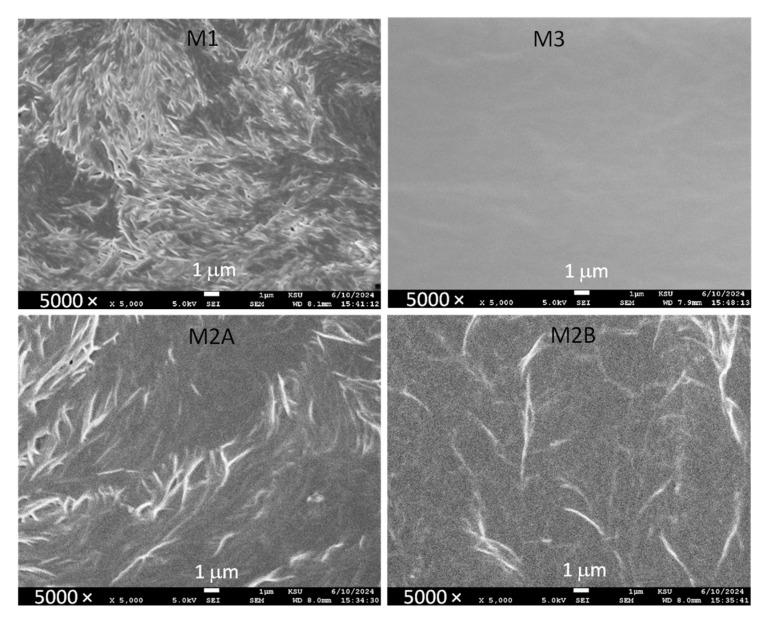
SEM images of solid redox mediators M1, M2A, M2B, and M3 at 25 °C. Scale bar 1 μm.

Figure 16 displays scanning electron microscopy images of M1, M2A, M2B, and M3. As mentioned earlier and demonstrated by polarized optical microscopy and DSC [25,26,27,29,30,31,32,33], the pure PEO-based redox mediators are known to exhibit high crystallinity. M1 displayed a fibril-like structure on its surface, suggesting the presence of semi-crystalline polymeric chains, which resulted in a comparatively low conductivity of the redox mediator [32]. The fibril-like structure was significantly diminished for M2A and M2B. The reduction is due to the waxy and plasticizing characteristics of succinonitrile, which lower the crystallinity of PEO and thereby enhance electrical conductivity. The waxy properties of succinonitrile are apparent in the SEM image of M3, which exhibited a smooth surface. The surface roughness hierarchy is M1 > M2A ≈ M2B > M3, indicating the efficacy of succinonitrile’s waxy characteristics.

**Figure 17:**
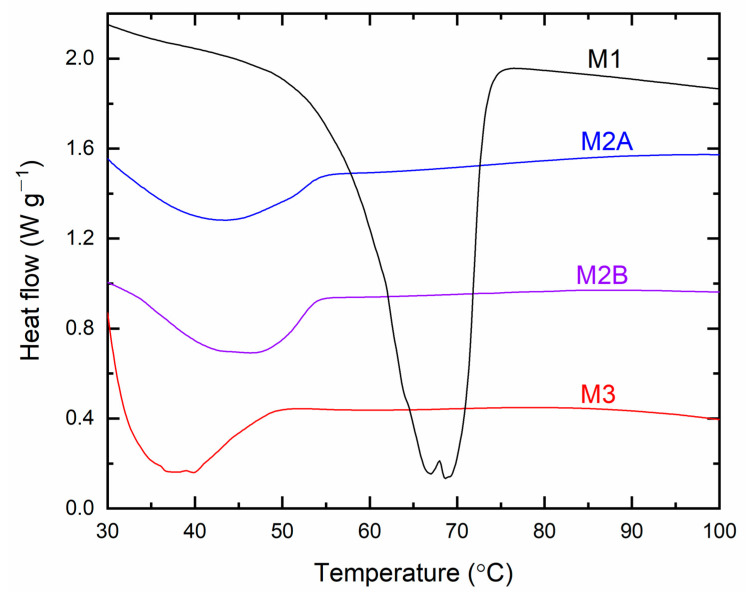
DSC curves for solid redox mediators M1, M2A, M2B, and M3.

Figure 17 presents the DSC curves for the solid redox mediators M1, M2A, M2B, and M3. The thermograms exhibited endothermic peaks that correspond to the melting temperature (*T*_m_) of SRMs [31,32,33]. M1 displayed a thermogram onset at approximately 47 °C, accompanied by doublet peaks at 66.9 °C and 68.8 °C. The doublet indicated a solid solution-type scenario in M1 [31,32,33,37], which reduced the crystallinity of PEO and subsequently enhanced the electrical conductivity compared to solid redox mediators prepared in the same manner with LiX (X = TFSI or CF_3_SO_3_) (cf. Table S1). M2A and M2B exhibited comparable thermograms, displaying shallow minima at 43.3 °C and 46.3 °C, respectively. M3 exhibited a deeper peak at 37.7 °C, attributed to the plastic crystal characteristics of the matrix (succinonitrile) [37]. The DSC curves indicated that the sequence of *T*_m_-values for the SRMs is M1 > M2B > M2A > M3, contrasting with the order of conductivity at 25 °C. The *T*_m_’s peak area for PEO-based redox mediators indicates the crystallinity of PEO [33]. The assessed values are 21.2 for M1, 2.5 for M2A, and 3 for M2B, demonstrating the order M1 > M2B > M2A. The area of *T*_m_’s peak for M2 is comparable with those prepared identically with LiX (X = TFSI or CF_3_SO_3_) [33].

**Figure 18:**
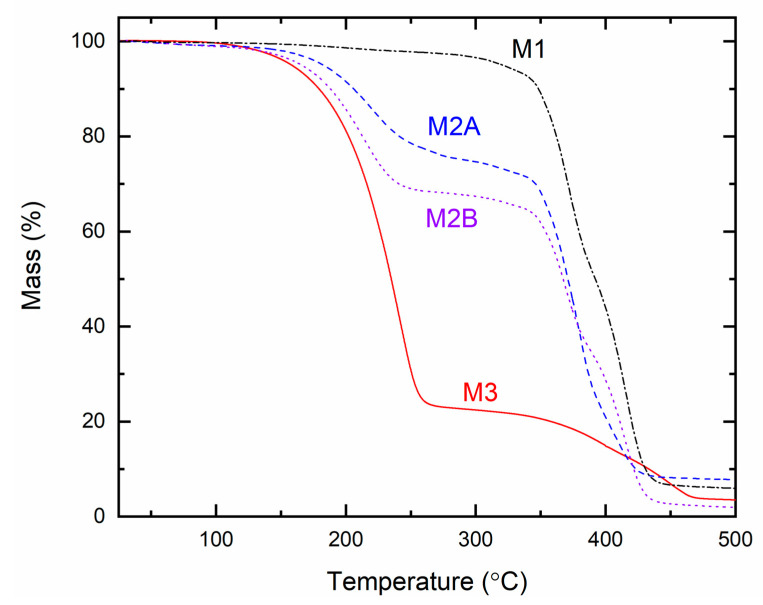
TGA curves for solid redox mediators M1, M2A, M2B, and M3.

Figure 18 illustrates the TGA curves for the solid redox mediators M1, M2A, M2B, and M3. The initial plateau region indicates the thermal stability of a polymer [31,32]. The curves exhibited initial flatness up to 200 °C for M1, 125 °C for M2A and M2B, and 100 °C for M3, comparable to the SRMs prepared in the same manner with LiX (X = TFSI or CF_3_SO_3_) [31,32]. M1 and M3, containing a single organic matrix of PEO and SN, respectively, demonstrated a single-stage decomposition of the organic substance [30,52,53]. In contrast, M2A and M2B exhibited double-stage decomposition due to their organic constituents, PEO and SN [30,52,53]. M2A (EO/Li^+^ = 112.9) exhibited a slower decomposition compared to M2B (EO/Li^+^ = 225.8), attributed to the higher concentration of ionic salts in M2A (cf. Table S3) [30,33]. This figure indicated the thermal stability order as M1 > M2A ≈ M2B > M3.

**Figure 19:**
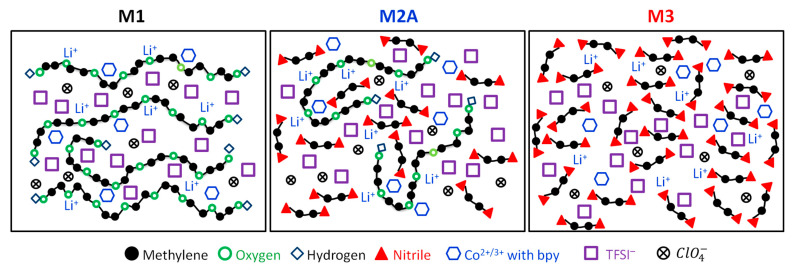
Schematic structure of solid redox mediators M1, M2A, and M3.

Based on the findings from electrical, structural, and thermal studies, a schematic structure of M1, M2A, and M3 can be illustrated (Figure 19). M1 displayed a combination of linear and entangled PEO chains, along with anions and cations. The presence of large cations and cobalt ions with bpy serves to function as a plasticizer, thereby reducing the crystallinity of PEO. The migration of lithium and cobalt ions through the ethereal oxygen of polymeric chains resulted in an electrical conductivity of 3 × 10^−5^ S cm^−1^ at 25 °C. M2A illustrated the entanglement of PEO chains along with the anionic and cationic components of salts. Entanglement occurred due to succinonitrile, alongside the plasticizing effects of TFSI^−^, ${ClO}_{4}^{-}$, and cobalt ions with bpy. This led to an improvement in electrical conductivity to 4 × 10^−4^ S cm^−1^. As noted earlier, succinonitrile serves as a solid solvent that facilitates the dissociation of ionic salts. The schematic structure for M3 illustrates that cations migrate freely through the cyano radicals of succinonitrile, resulting in an electrical conductivity of 2 × 10^−3^ S cm^−1^ at 25 °C.

## 4. Conclusions

The key findings from the IS, XRD, FT-IR spectroscopy, XPS, SEM, DSC, and TGA studies are summarized as follows.

(a) The Bode and Nyquist curves, XPS spectra, and SEM images of M2 exhibited similarities to those of M1, revealing two distinctive groups of solid redox mediators, PEO-like and SN-like mediators.
(b) The Bode and Nyquist curves of M1 and M2 showed R_1_ − C circuit-type patterns, whereas M3 portrayed R_1_ − (R_2_‖C) circuit-type patterns.
(c) The Bode (phase angle) plots at 25 °C demonstrated the negative-slope ramp for M2 nearly at the middle of the ramps for M1 and M3. The FT-IR spectra of M2 displayed a spectrum characteristic of the PEO–SN blend. Both plots indicated a composite electrolyte behavior of M2.
(d) The electrical conductivity at 25 °C had the following sequence: M1 < M2B < M2A < M3. M2 had the lowest activation energy in the solid-state region.
(e) M2 depicted no peaks in the XRD patterns, whereas M1 and M3 showed weak characteristic peaks of the matrix.
(f) The FT-IR spectroscopy and XPS results demonstrated the matrix–salt interaction hierarchy as M1 > M2B > M2A > M3.
(g) The *T*_m_-value and its corresponding area had the following order: M1 > M2B > M2A > M3.
(h) The thermal stability followed the order of M1 > M2 > M3, with M2 at ~125 °C.

These findings are well corroborated by the results of solid ${Co}^{2+}/{Co}^{3+}$ redox mediators prepared identically with LiX (X = TFSI or CF_3_SO_3_). These findings also established that the PEO–SN blend is an excellent choice as a solid matrix for achieving electrical conductivity of more than 10^−4^ S cm^−1^ at 25 °C with activation energy below 0.1 eV to ensure optimal performance of a device.

## Figures and Tables

**Table 1:** Electrical transport parameters of solid redox mediators M1, M2A, M2B, and M3. (I) and (II) stand for temperature regions. Please see the text for details.

Redox Mediators	σ_25°C_ (S cm^−1^)	$Log\sigma -T^{-1}$ Curve’s Nature	*E*_a_ (eV)	*σ_o_*(S cm^−1^)
M1	2.98 × 10^−5^	Arrhenius	0.99 (I), 0.2 (II)	2.12 × 10^12^ (I), 1.62 (II)
M2A	3.9 × 10^−4^	VTF	0.04	1.26
M2B	1.47 × 10^−4^	VTF	0.04	2.23
M3	2.05 × 10^−3^	Arrhenius	0.74 (I), 0.17 (II)	6 × 10^9^ (I), 4.5 (II)

## Data Availability

The original contributions presented in this study are included in the article/Supplementary Material. Further inquiries can be directed to the corresponding author.

## Supplementary Materials

The following supporting information can be downloaded at: https://www.mdpi.com/article/10.3390/polym18010142/s1, Figure S1: Chemical structure of ingredients; Figure S2: FT-IR spectra in the fingerprint region for ionic salts; Figure S3: Best fit of XPS spectrum for S 2p element of solid redox mediators; Figure S4: Best fit of XPS spectrum for Cl 2s element of solid redox mediators; Figure S5: Best fit of XPS spectrum for C 1s element of solid redox mediators; Figure S6: Best fit of XPS spectrum for N 1s element of solid redox mediators; Figure S7: Best fit of XPS spectrum for O 1s element of solid redox mediators; Figure S8: Best fit of XPS spectrum for F 1s element of solid redox mediators; Table S1: Electrical conductivity ($\sigma_{25{}^{\circ}C}$) and pseudo-activation energy (E_a_) of the [poly(ethylene oxide)-succinonitrile] blend-based solid $I^{-}/I_{3}^{-}$ and ${Co}^{2+}/{Co}^{3+}$ redox mediators; Table S2: Chemicals used for the synthesis of redox mediators; Table S3: Composition of solid Co(II/III) redox mediators; Table S4: Details of characterization techniques, along with the equipment used; Table S5: Observed vibrational frequencies (in cm^−1^) of solid redox mediators, solid matrix, and ionic salts.

## Abbreviations

The following abbreviations are used in this manuscript:

DSSC	Dye-sensitized solar cell
SRM	Solid redox mediator
PEO	Poly(ethylene oxide)
SN	Succinonitrile
bpy	tris-(2,2′-bipyridine)
TFSI	bis(trifluoromethyl) sulfonylimide
CF_3_SO_3_	Triflate, trifluoromethanesulfonate
ClO_4_	Perchlorate
IS	Impedance spectroscopy
FT-IR	Fourier-transform infrared spectroscopy
XRD	X-ray diffractometry
XPS	X-ray photoelectron spectroscopy
SEM	Scanning electron microscopy
DSC	Differential scanning calorimetry
TGA	Thermogravimetric analysis
σ	Electrical conductivity
E_a_	Activation energy
σ_o_	Pre-exponential factor
k_B_	Boltzmann constant
T_o_	Free-volume temperature
T_m_	Melting temperature
R = Intensity/width	where width is full width at half maximum
ΔP	The position of the element of M2A, M2B, or M3 relative to that of M1
