# Supplementary material for: Impedance Spectroscopy Study of Solid Co(II/III) Redox Mediators Prepared with Poly(Ethylene Oxide), Succinonitrile, Cobalt Salts, and Lithium Perchlorate for Dye-Sensitized Solar Cells
Source: Polymers (Basel). 2026 Jan 4;18(1):142. doi: 10.3390/polym18010142 (PMC12787447; doi:10.3390/polym18010142)
Supplement: Supplementary file 1 [file polymers-18-00142-s001.zip › polymers-3937108-supplementary.pdf]

Impedance Spectroscopy Study of Solid Co(II/III) Redox Mediators Prepared with Poly(Ethylene Oxide), Succinonitrile, Cobalt Salts, and Lithium Perchlorate for Dye-Sensitized Solar Cells

Ravindra Kumar Gupta,[1,*] Ahamad Imran,[1] Aslam Khan,[1] Muhammad Ali Shar,[1] Khalid M. Alotaibi,[1,2] Idriss Bedja,[3] Abdullah Saleh Aldwayyan [4]

[1] King Abdullah Institute for Nanotechnology, King Saud University, Riyadh 11451, Saudi Arabia; rgupta@ksu.edu.sa; aimran@ksu.edu.sa; aslamkhan@ksu.edu.sa; mashar@ksu.edu.sa

[2] Department of Chemistry, College of Science, King Saud University, Riyadh 11451, Saudi Arabia; khalid.m@ksu.edu.sa

[3] Department of Optometry, College of Applied Medical Sciences, King Saud University, Riyadh 11433, Saudi Arabia; bedja@ksu.edu.sa

[4] Department of Physics and Astronomy, College of Science, King Saud University, Riyadh 11451, Saudi Arabia; dwayyan@ksu.edu.sa

Supplementary Information

Table S1. Electrical conductivity ($\sigma_{25°C}$) and pseudo-activation energy (E_a) of the [poly(ethylene oxide)–succinonitrile] blend-based solid I^-/I_3^- and Co^{2+}/Co^{3+} redox mediators. These mediators exhibit the Vogel–Tamman–Fulcher-type behavior.

**Redox couple**	**Ionic salts* and dispersoid**	**Ethylene oxide/ M$^+$ (M = Li or K)**	**$\sigma_{25°C}$ (S cm^{-1})**	**E_a (eV)**
I^-/I_3^-	LiI, I_2	8	3×10^{-4}	0.08
I^-/I_3^-	LiI, I_2, laponite	8	4.9×10^{-4}	0.086
I^-/I_3^-	KI, I_2	11.9	7×10^{-4}	0.086
Co^{2+}/Co^{3+}	Co[bpy]$_3$(TFSI)$_2$, Co[bpy]$_3$(TFSI)$_3$, LiTFSI	113	4.3×10^{-4}	0.06
Co^{2+}/Co^{3+}	Co[bpy]$_3$(TFSI)$_2$, Co[bpy]$_3$(TFSI)$_3$, LiTFSI	226	7.2×10^{-4}	0.05
Co^{2+}/Co^{3+}	Co[bpy]$_3$(TFSI)$_2$, Co[bpy]$_3$(TFSI)$_3$, $LiCF_3SO_3$	108.4	4.7×10^{-4}	0.06
Co^{2+}/Co^{3+}	Co[bpy]$_3$(TFSI)$_2$, Co[bpy]$_3$(TFSI)$_3$, $LiCF_3SO_3$	216.8	3.1×10^{-4}	0.06

* bpy, tris-(2,2'-bipyridine); TFSI, bis(trifluoromethyl) sulfonylimide

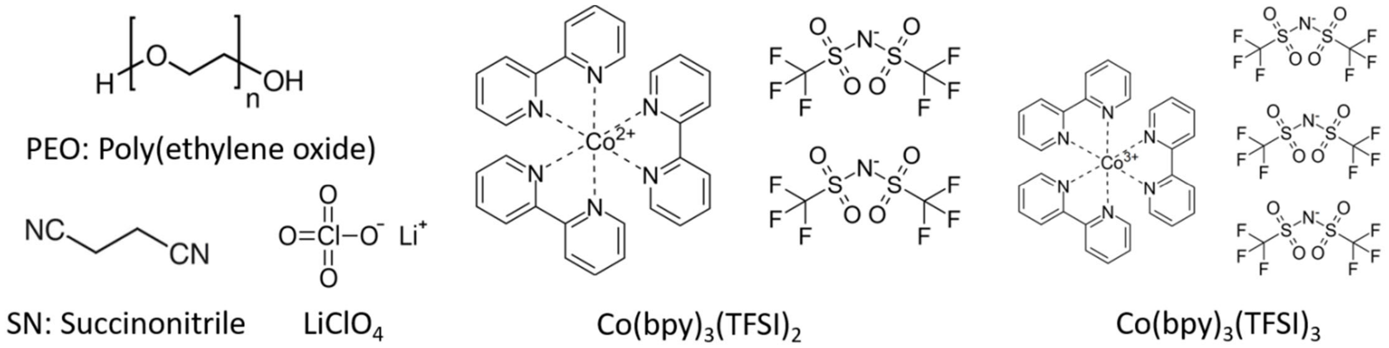

Figure S1. Chemical structure of ingredients.

Table S2. Chemicals used for the synthesis of redox mediators.

Chemicals	Purity (%)	Make
Acetonitrile	99.9	Fisher Scientific, USA
Poly(ethylene oxide)	99	Sigma-Aldrich, Inc., USA
Succinonitrile	99	Sigma-Aldrich, Inc., USA
$LiClO_4$	99.99	Sigma-Aldrich, Inc., USA
$Co(bpy)_3(TFSI)_2$	95	Dyenamo, Sweden
$Co(bpy)_3(TFSI)_3$	98	Dyenamo, Sweden

Table S3. Composition of solid Co(II/III) redox mediators.

Redox mediators	EO/Li^+	Poly(ethylene oxide) (g)	Succinonitrile (g)	$Co(bpy)_3(TFSI)_2$ (g)	$Co(bpy)_3(TFSI)_3$ (g)	$LiClO_4$ (g)
M1	225.8	0.5600	-	0.1531	0.0462	0.0060
M2A	112.9	0.2800	0.2800	0.1531	0.0462	0.0060
M2B	225.8	0.5600	0.5600	0.1531	0.0462	0.0060
M3	-	-	0.5600	0.1531	0.0462	0.0060

Table S4. Details of characterization techniques, along with the equipment used.

Technique	Methodology
IS	This method is utilized to assess the electrical conductivity of an electrolyte. The assessment of electrical conductivity in relation to temperature is essential for determining the activation energy. An electrolyte film was positioned between the plates of a sample holder. The plate was composed of either stainless steel or platinum, serving as a blocking electrode. The measured thicknesses of the M1, M2, and M3 films were approximately 0.5 mm, 0.3 mm, and 0.3 mm, respectively. The areas measured were 0.38, 0.5, and 0.16 cm^2, respectively. The electrolyte film underwent a 20 mV AC voltage application with a frequency range 10^6 to 1 Hz for M1 and M2, and 10^6 to 10^{-1} Hz for M3. The measurement conducted using a Palmsens impedance analyser (model PalmSens4, Houten, The Netherlands) yielded Bode plots illustrating phase angle and impedance with frequency, which subsequently produced a complex impedance plot (Nyquist plot). The Nyquist plot typically features a semicircle in the high-frequency region, along with a straight line in the intermediate- and low-frequency regions. The intercept of the semicircle in the high-frequency domain results in bulk resistance, which in turn produces the electrical conductivity.
XRD	A cover glass with an area of 1 cm^2 was coated with a substantial layer of the electrolyte. A CuKα line X-ray beam (1.54184 Å) was directed onto the film within a range of 10–80° at increments of 0.06°, utilizing a Bruker X-ray diffractometer (model D2 Phaser, Karlsruhe, Germany).
FT-IR	A thin film of electrolyte was formed on a KBr pellet with a diameter of 2.5 cm and a thickness of 0.2 cm. The FT-IR spectrum of this film was collected from 4000 to 400 cm^{-1} with a resolution of 1 cm^{-1} at 25 °C using a Perkin Elmer FT-IR spectrometer (model Spectrum 100, Waltham, USA).
XPS	A mesoporous layer of titania with an area of 1 cm^2 and a thickness of 7 μm on a microscopic glass surface was prepared using Dyesol's 18-NRT titania paste by the doctor blade method, followed by sintering it at 500°C. This mesoporous film was infiltrated with a solid redox mediator. This film was exposed to MgK*α* X-ray radiation (1253.6 eV) at ultra-high vacuum conditions utilizing a JEOL JPS-9030 Photoelectron Spectrometer (Tokyo, Japan). The film was scanned four times, from 1000 to 0 eV with a step of 1 eV to look at the elements. Scanning five times at a step of 0.1 eV for 100 ms provided a precise spectrum of the element. The spectrum was smoothed for 9 points along with the rubberband baseline using the OPUS software. The spectra of the elements were corrected with the standard C 1s peak at 284.6 eV. The spectrum was fitted to determine the position, intensity, and width (full width at half maximum) of the peak. To make the ratio (R) dimensionless, the peak's intensity was divided by its width.
SEM	An electrolyte film was created on a mesoporous TiO_2 layer with a thickness of 7 μm, situated on a microscopic glass surface measuring 1 cm^2. The glass has been fragmented into numerous small shards. A

	tiny section of this film underwent platinum coating with a JEOL JFC-1600 auto fine coater (Tokyo, Japan), followed by imaging with a JEOL scanning electron microscope (model JSM-7600F, Tokyo, Japan).
DSC	An aluminum crucible was sealed, containing approximately 1-2 mg of electrolyte. The crucible underwent heat flow measurement ranging from 25°C to 100°C at a rate of 10°C per minute, utilizing a Mettler-Toledo unit (model TGA/DSC1, Schwerzenbach, Switzerland) within a N_2 gas atmosphere.
TGA	An alumina pan containing 5–10 mg of electrolyte was employed in a Mettler-Toledo unit (model TGA/DSC1, Schwerzenbach, Switzerland) under a nitrogen gas atmosphere to evaluate weight loss from room temperature to 600°C at a heating rate of 10°C per minute.

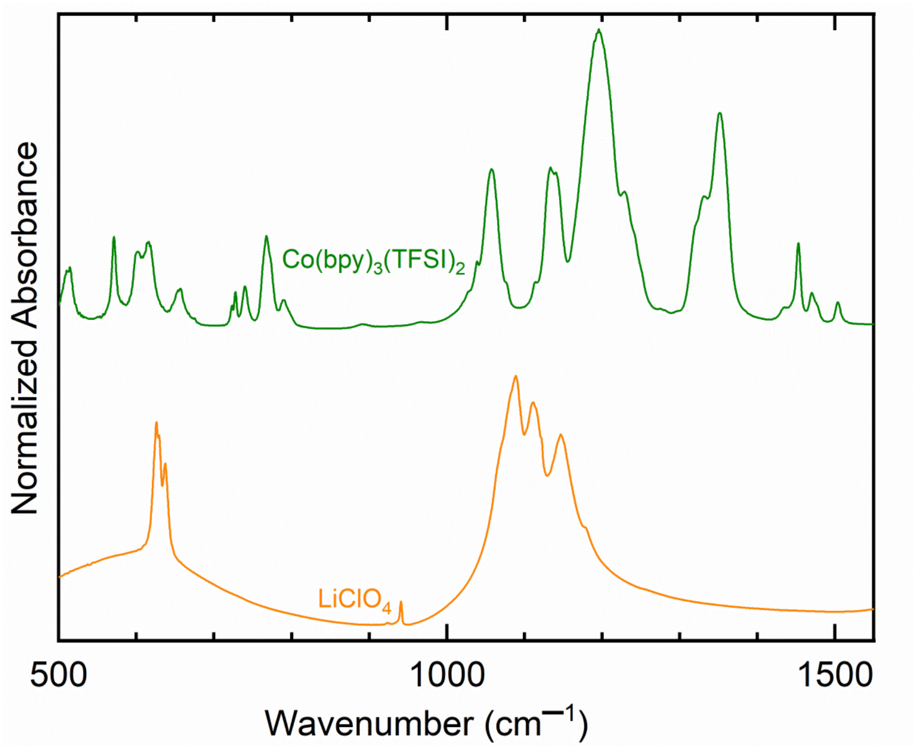

Figure S2. FT-IR spectra in the fingerprint region for ionic salts. Both cobalt salts exhibited a similar pattern.

Table S5. Observed vibrational frequencies (in cm^{-1}) of solid redox mediators M1, M2A, M2B, and M3. This also included vibrational frequencies of the solid matrix (PEO, SN-PEO blend, and SN), and ionic salts ($Co(bpy)_3(TFSI)_2$ and $LiClO_4$) for comparative analysis.

**PEO**†	**M1**	**Blend**	**M2A**	**M2B**	**SN**	**M3**	**Co salt**	**$LiClO_4$**	**Assignment**‡
	513w		514m	514m		514m	514m		$\delta_{a,SO3}$
	570m		570m	571m		571m	571s		$\delta_{a,SO3}$
	600sh	604m	604sh	603sh	604s	603m	601m		δ_{CCC}, $\nu_{a,CF3}$
	618m		618m	617m		618m	616m	626s	o.p.ring, ν_{ClO4}(free)
	652w		652w	653w		653m	656m	637sh	$\delta_{a,SO3}$, ν_{ClO4}(pair)
	738w		738m	738w		738m	740m		$\nu_{a,C-CN}$,$\nu_{s,SNS}$
	780m	762m	***780m***	***780m***	762s	***769s***	767s		δ_{CH2}, ring
			786sh			789m	789m		$\nu_{a,SNS}$
		819w			819s	819w			ν_{C-CN}
843s	842m	846m	845m	843m					$\rho_{a,CH2}$, ν_{CO}
		918sh			918s	918w			$\nu_{s,C-CN}$
963s	964m	953s	953m	954m	963s	963m			$\rho_{a,CH2}$, t_{CH2}, ν_{C-CN}
		1002m			1002s	1002m			ρ_{CH2}
1061m	1061sh		1060sh	1060sh		1059s	1057s	1089s	$\nu_{a,COC}$, $\rho_{a,CH2}$, $\nu_{a,SNS}$, ν_{ClO4}
1109s	**1113s**	1105s	**1098s**	**1108s**				1111sh	$\nu_{s,COC}$,ν_{ClO4}
1149s	**1144sh**		1133sh	1131sh		1137s	1133s	1147sh	ν_{CC},$\nu_{s,SO2}$,$\nu_{a,CF3}$, ν_{ClO4}
	1194m	1196w	1196s	1195s	1199m	1197s	1196s		t_{CH2}, $\nu_{a,CF3}$
			1227m	1227w	1233s	1228sh	1229sh		t_{CH2}, i.p.ring, $\nu_{s,CF3}$
1242m	1242w	1251m	1247w	1250w					$t_{a,CH2}$
1280m	1280w	1299m							$t_{a,CH2}$, $t_{s,CH2}$
			1334sh	1334sh	1337s	1337sh	1331sh		ω_{CH2}, $\nu_{a,SO2}$
1342s	1343m	1350m	1353s	1353s		1353s	1351s		$\omega_{a,CH2}$, $\nu_{a,SO2}$
		1426s			1426s	1426s			δ_{CH2}
1454m	***1442m***	1453w	***1441m***	***1443m***		***1443sh***	1453m		$\delta_{a,CH2}$, ring
1467m	1467m	1469w	1471m	1467m		***1474m***	1470m		$\delta_{a,CH2}$, ring
		2251s	2253m	2253m	2254s	2254s			$\nu_{s,C\equiv N}$
2861sh		2875s	**2881s**	**2876s**	2952s	2951m			$\nu_{s,CH2}$
2889s	**2884s**	2899s			2989s	2989s			$\nu_{a,CH2}$

† Notation for relative intensity: w (weak), m (medium), s (strong), and sh (shoulder).

‡ Notation for modes: stretching (ν), bending (δ), wagging (ω), twisting (t), rocking (ρ), symmetric (s), asymmetric (a), in-plane (i.p.), and out-of-plane (o.p.).

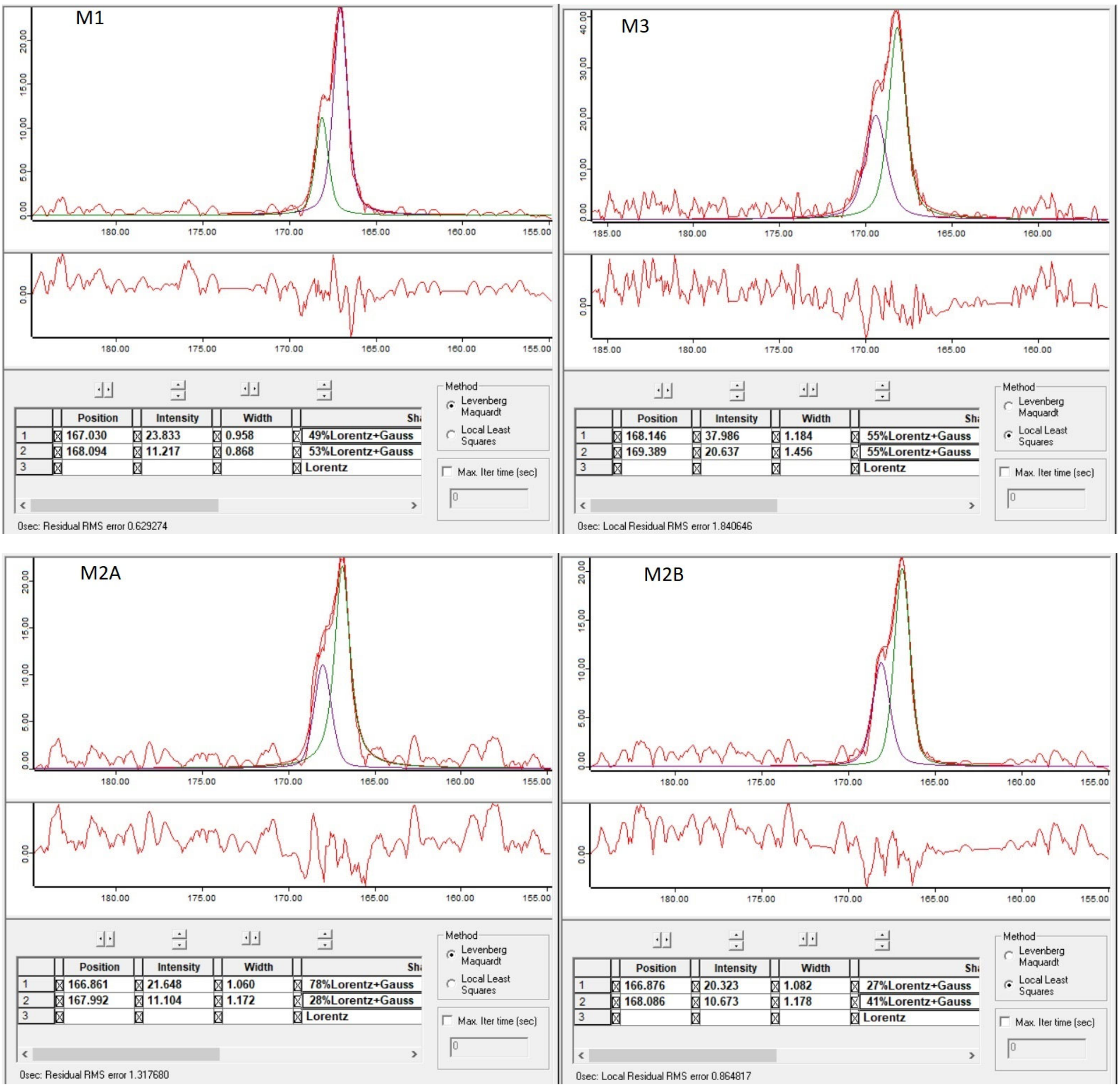

Figure S3. Best fit of XPS spectrum for S 2p element of solid redox mediators M1, M2A, M2B, and M3. These spectra portrayed a small S 2p peak because of the –SO_2– group of TFSI at ≈167 eV for the spin of 3/2, associated with a shoulder peak at ≈168.1 eV for the spin of ½.

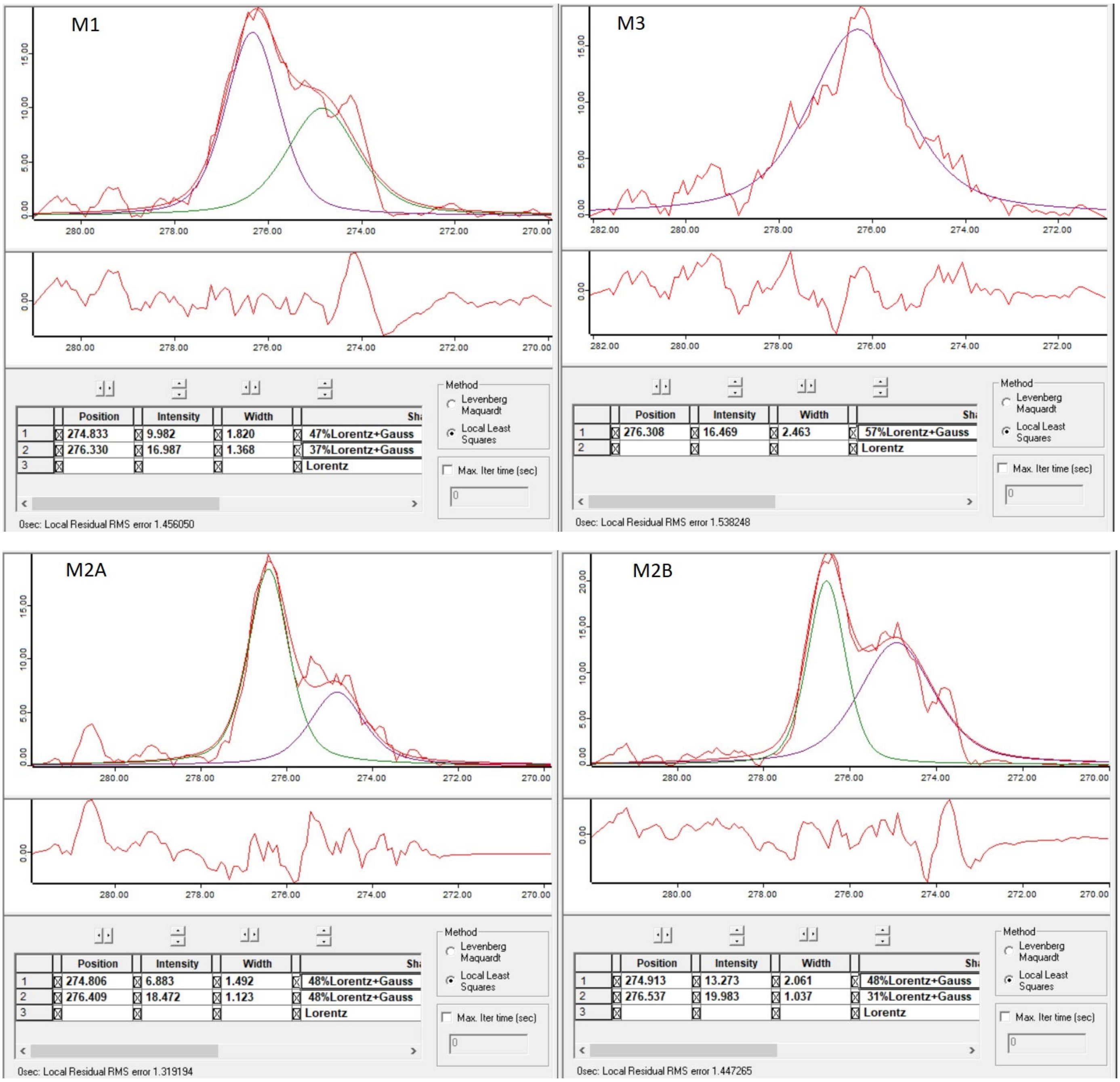

Figure S4. Best fit of XPS spectrum for Cl 2s element of solid redox mediators M1, M2A, M2B, and M3. The Cl 2s peak appeared at ≈276.3 eV due to the ClO_4^- ions. M1, M2A, and M2B portrayed a C 1s satellite peak nearly at 274.8 due to excess hydrocarbons in PEO.

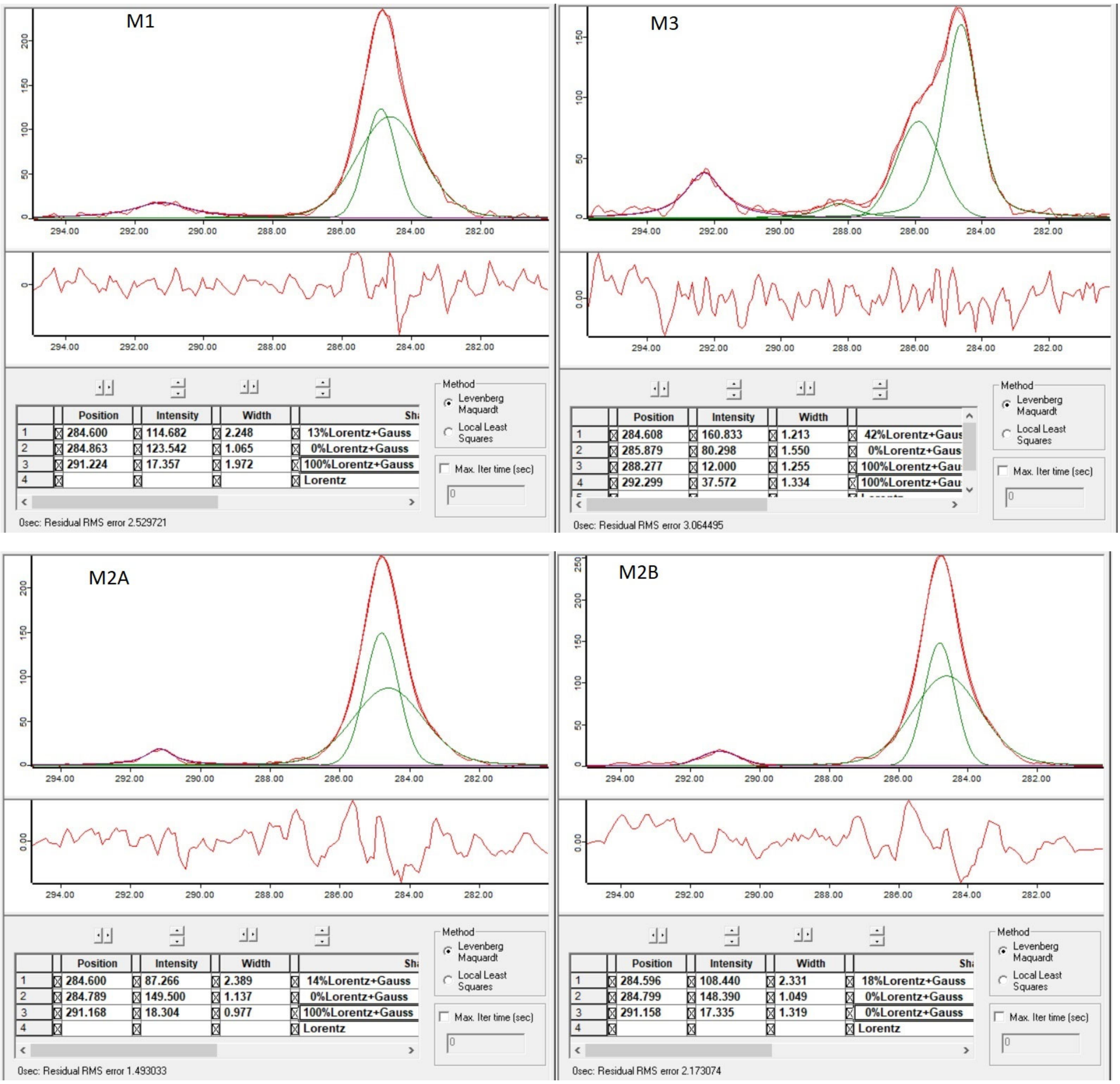

Figure S5. Best fit of XPS spectrum for C 1s element of solid redox mediators M1, M2A, M2B, and M3. These spectra exhibited a standard peak at 284.6 eV for the alkyl (–C–C–) group associated with a peak at ≈284.9 eV for the bpy's ring or –C–C–O– group, a small peak at ≈288.2 eV for the –C≡N group, and a distinctive small peak at ≈291.2 eV for the –CF_3 group.

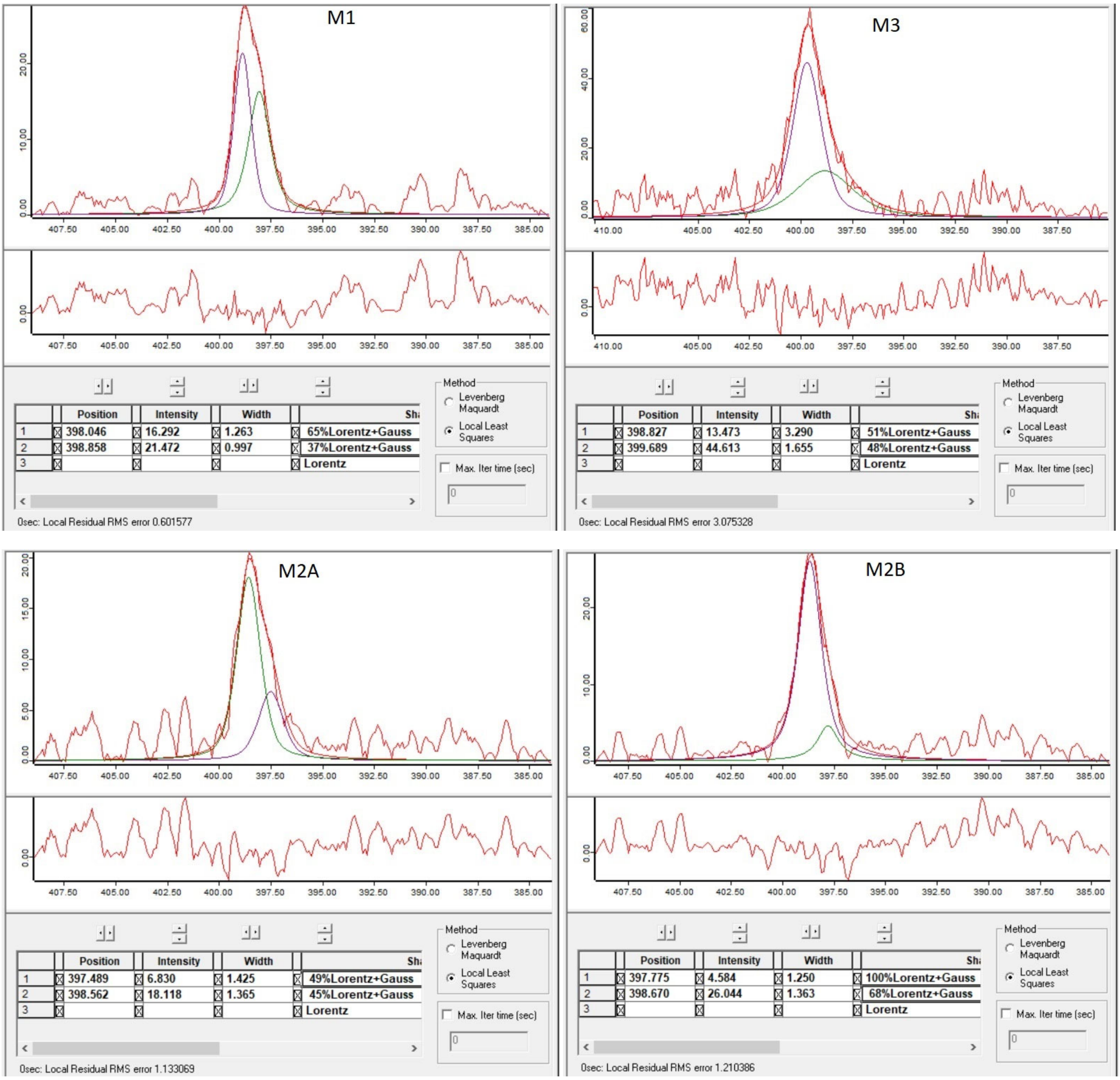

Figure S6. Best fit of XPS spectrum for N 1s element of solid redox mediators M1, M2A, M2B, and M3. The small-heighted peak consisted of two deconvoluted peaks at ≈398 eV and ≈398.9 eV. The source of N 1s is the bpy and TFSI ion as well as the –C≡N group in succinonitrile.

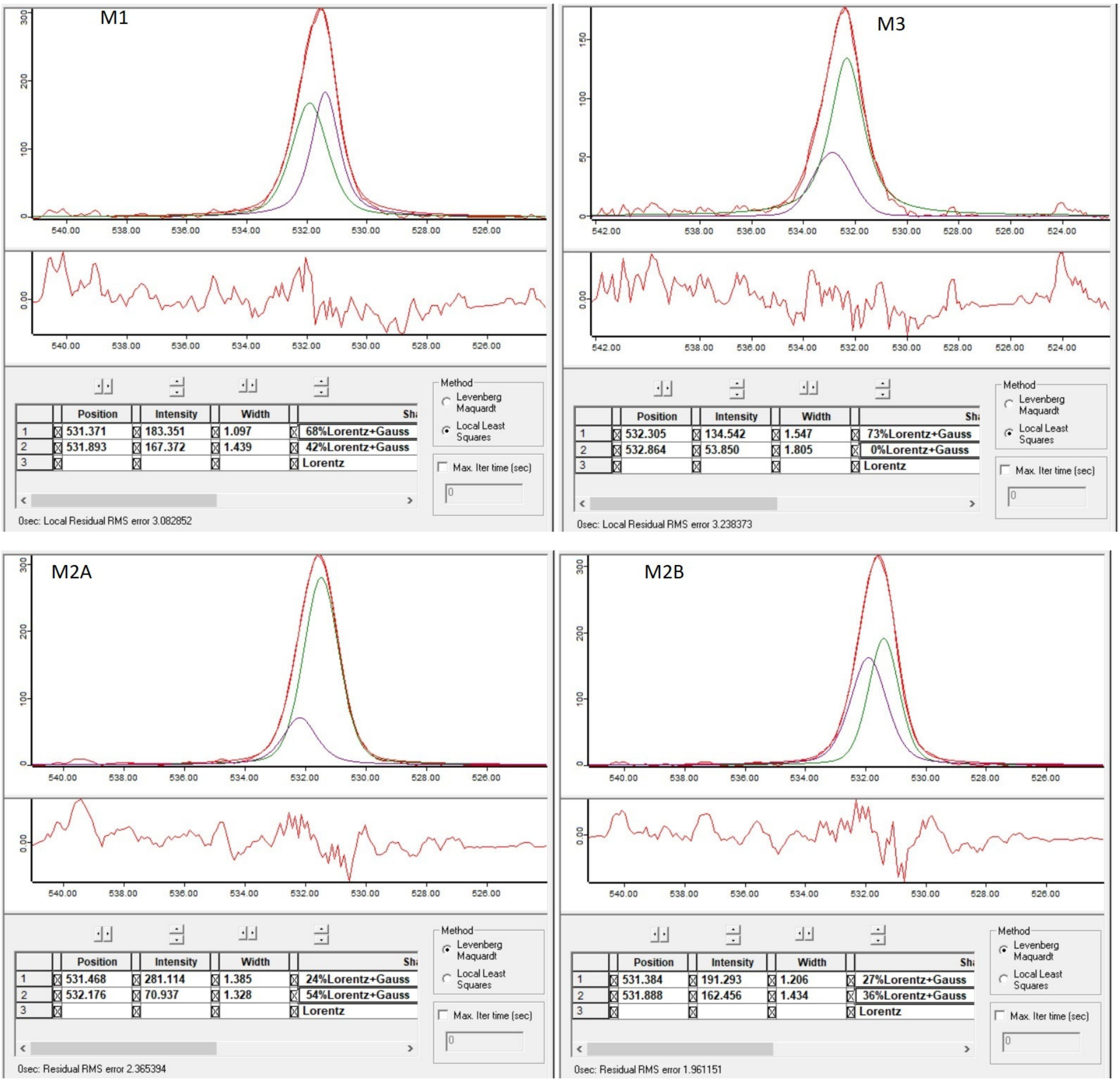

Figure S7. Best fit of XPS spectrum for O 1s element of solid redox mediators M1, M2A, M2B, and M3. The strong O 1s peak appeared at around 531.4 eV and 531.9 eV because of the spin-orbit coupling phenomenon. The source of O 1s is the –C–C–O–, $–SO_2–$, and ClO_4 groups.

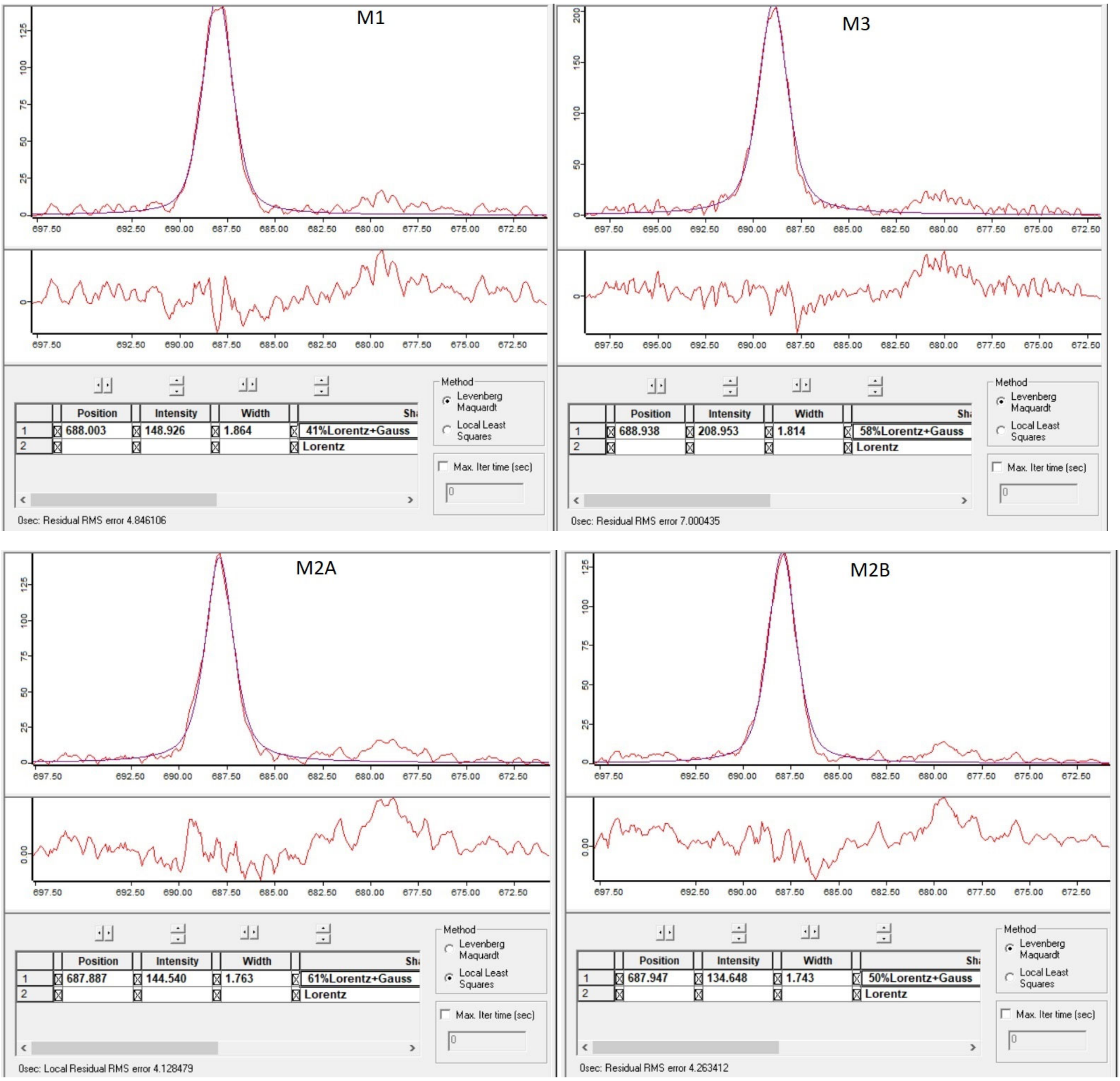

Figure S8. Best fit of XPS spectrum for F 1s element of solid redox mediators M1, M2A, M2B, and M3. This peak appeared at ≈688 eV due to the –CF_3 group of the TFSI ion.
